# Aversive Behavior in the Nematode *C*. *elegans* Is Modulated by cGMP and a Neuronal Gap Junction Network

**DOI:** 10.1371/journal.pgen.1006153

**Published:** 2016-07-26

**Authors:** Michelle C. Krzyzanowski, Sarah Woldemariam, Jordan F. Wood, Aditi H. Chaubey, Chantal Brueggemann, Alexander Bowitch, Mary Bethke, Noelle D. L’Etoile, Denise M. Ferkey

**Affiliations:** 1 Department of Biological Sciences, University at Buffalo, The State University of New York, Buffalo, New York, United States of America; 2 Department of Cell and Tissue Biology, University of California, San Francisco, San Francisco, California, United States of America; University of California San Diego, UNITED STATES

## Abstract

All animals rely on their ability to sense and respond to their environment to survive. However, the suitability of a behavioral response is context-dependent, and must reflect both an animal’s life history and its present internal state. Based on the integration of these variables, an animal’s needs can be prioritized to optimize survival strategies. Nociceptive sensory systems detect harmful stimuli and allow for the initiation of protective behavioral responses. The polymodal ASH sensory neurons are the primary nociceptors in *C*. *elegans*. We show here that the guanylyl cyclase ODR-1 functions non-cell-autonomously to downregulate ASH-mediated aversive behaviors and that ectopic cGMP generation in ASH is sufficient to dampen ASH sensitivity. We define a gap junction neural network that regulates nociception and propose that decentralized regulation of ASH signaling can allow for rapid correlation between an animal’s internal state and its behavioral output, lending modulatory flexibility to this hard-wired nociceptive neural circuit.

## Author Summary

Studying the logic of small neural circuits is an essential step toward understanding more complex circuits and, ultimately, the computational and integrative properties of whole nervous systems. With a compact nervous system (just 302 neurons) and a well-characterized behavioral repertoire, the small roundworm *C*. *elegans* serves as an excellent animal model to study circuit-level modulation of neuronal function. By employing a combination of genetic and behavioral approaches, we have identified a non-cell-autonomous role for the guanylyl cyclase ODR-1 in the regulation of nociceptive sensory behaviors, and we provide the first evidence for circuit-level modulation of neuronal activity by the second messenger cGMP. While revealing a new mechanism for the coordination and optimization of animal behavior, our work also supports a growing body of evidence that hard-wired neuronal circuitry can be dynamically repurposed in a context-dependent manner.

## Introduction

Chemical stimuli, including odorants and tastants, can provide information about food availability and quality to affect appetitive behaviors across species. While all sensory circuits use specialized cells in the periphery to detect environmental stimuli, how the sensitivity of sensory neurons is tuned and how chemical information is processed and relayed through downstream interneurons remains largely unknown. As ultrastructural analyses of simple brains and small brain regions are providing connectome data [1–7], the challenge that lies ahead is in understanding the dynamic properties of circuitry usage. While mapped physical connections show the potential for information flow, the breadth of possibilities must also be reconciled with a circuit’s potential for neuromodulation as animals interact with a complex and changing environment. For example, studies in systems ranging from invertebrates to mammals have revealed that chemosensory responses and feeding behaviors are modulated by an animal’s nutritional state [8–16]. Furthermore, there is incredible complexity in the mechanisms by which nutritional status ultimately modulates chemosensory and feeding behaviors, reflecting the nervous system’s need to integrate information about what an animal has eaten, how much and when. For example, neuropeptides, neurotransmitters (e.g. serotonin and dopamine), hormones (e.g. insulin) and metabolites have all been shown to modulate chemosensory responses [8,12,15,17–21].

Chemical synapses allow neurons to communicate with each other through the vesicular release of neurotransmitters into synaptic clefts between the cells. These molecules then bind to receptors on the postsynaptic neurons. In contrast, gap junctions allow for direct cytoplasmic communication and electrical coupling between neurons. As such, gap junctions are often referred to as electrical synapses. Importantly, the presence of gap junctions in the nervous system allows for the establishment of even more complex circuits than can be generated by synaptic signaling alone.

Vertebrate gap junctions are formed through the association of transmembrane connexin proteins. Within one cell, six connexin proteins assemble to form one connexon hemichannel. Two hemichannels on neighboring cells then dock which each other, allowing homotypic (consisting of a single protein species on both cell membranes), heterotypic (consisting of two different homomeric connexons on the two cell membranes) or heteromeric (consisting of a mixture of protein species on both cell membranes) gap junctions to be made [22,23]. Gap junction communication allows for the transmission of action potentials [24,25], diffusion of metabolites and nutrients [26] and diffusion of second messengers, including Ca^2+^, IP_3_ and the cyclic nucleotides cAMP and cGMP [27–32]. The ability to pass such a diversity of molecules affords them the potential to repurpose hardwired circuitry to modify responses to diverse environmental stimuli.

The gap junction protein family consists of vertebrate connexins and invertebrate innexins (invertebrate analogues of the connexins) [33,34]. The *C*. *elegans* genome encodes 25 members of this protein family [35]. Although the functions of many *C*. *elegans* innexins remain unknown, the characterized cases have shown that these gap junction components are involved in diverse processes ranging from embryonic development and cell fate determination to adult neural functions [35–37].

*C*. *elegans* is an ideal model system in which to study the link between hardwired connectivity and the functional circuitry usage that drives animal behavior. The serial electron micrographs that showed the anatomical positioning of each of the 302 *C*. *elegans* hermaphrodite neurons also revealed all of the morphologically identifiable connections between neurons and between neurons and muscles [2,38–41]. The recently updated *C*. *elegans* wiring diagram shows a total of 6393 chemical synapses, 890 gap junctions and 1410 neuromuscular junctions [42]. It is through this interconnected neural network that *C*. *elegans* exploits a highly developed chemosensory system to detect olfactory and gustatory cues associated with food, danger and mating [43–45]. In general, animals move towards chemicals that indicate a favorable environment, such as a potential food source, and away from stimuli that suggest a harmful environment.

The 11 pairs of *C*. *elegans* head chemosensory neurons extend their ciliated ends to the tip of the animal’s nose, allowing for direct or indirect exposure to sensory stimuli in their environment [2,39,40]. Cellular laser ablation studies have been used to reveal the function of individual neuron pairs [43]. For example, the ASEs sense water-soluble attractants, while the AWA and AWC neurons detect volatile odorants that *C*. *elegans* are attracted to and chemotax towards. In addition, while the ASJ, ASI, ADF and ASG sensory neurons are primarily involved in regulating dauer formation, they do also play a role in other processes, including a minor role in chemotaxis. Conversely, the ASH, ADL, AWB and ASK chemosensory neurons detect aversive stimuli that animals avoid by initiating backward locomotion upon stimulus detection.

The two bilaterally symmetric ASH nociceptors are particularly important for the avoidance of noxious stimuli, as they are “polymodal.” This neuron pair responds to a broad range of aversive stimuli, including not only soluble chemicals (e.g. the bitter tastant quinine, heavy metals and SDS) and odorants (e.g. octanol), but also ions (e.g. Na+), osmotic stress and mechanosensory stimulation (nose touch) [46–54]. ASH activation elicits reversal and stimulus avoidance because these glutamatergic sensory neurons synapse onto command interneurons that drive backward locomotion via their connections with motor neurons. Thus, the nociceptive sensory system of *C*. *elegans* bears resemblance to its mammalian counterparts, in which noxious stimuli (including chemicals) are sensed predominantly by peripheral glutamatergic sensory neurons that synapse onto spinal dorsal horn neurons and, following further sensory processing in multiple regions of the brain, generate the perception of pain and aversive behavior [55–57].

We previously reported a role for the cGMP-dependent protein kinase EGL-4 in the modulation of ASH-evoked nociceptive behavioral responses [58]. *C*. *elegans* lacking EGL-4 function are hypersensitive in their response to a subset of ASH-detected stimuli; *egl-4(lof)* animals avoid dilute stimuli that wild-type animals do not respond to. Our data suggested that EGL-4 likely normally acts to dampen ASH sensitivity by phosphorylating and activating the GTPase activating proteins RGS-2 and RGS-3, which then downregulate G protein-coupled sensory signaling in the ASH nociceptors. Surprisingly, although EGL-4 requires cGMP binding to negatively regulate ASH sensitivity, no guanylyl cyclases are known to be expressed in ASH [59].

Herein we provide evidence that the *C*. *elegans* transmembrane guanylyl cyclase ODR-1 functions in a non-cell-autonomous manner to provide cGMP to regulate EGL-4 function in ASH. Like *egl-4(lof)* animals, *odr-1(lof)* animals are hypersensitive in their avoidance of a subset of ASH-detected stimuli. However, while EGL-4 functions directly in the ASHs, ODR-1 expression in the AWB, AWC and ASI head sensory neurons is sufficient to restore normal behavioral sensitivity to *odr-1(lof)* animals. We further provide evidence that the pool of cGMP produced by ODR-1 flows through a gap junction network from its site of production to the ASH nociceptors. Taken together, our data reveal a new way by which an animal’s nervous system can utilize information about the organism’s well-being to set the threshold of nociceptor sensitivity and coordinate behavioral responses that are appropriate for its internal state.

## Results

### ODR-1 Functions in Multiple Sensory Neurons to Dampen Quinine Sensitivity

**Fig 1:**
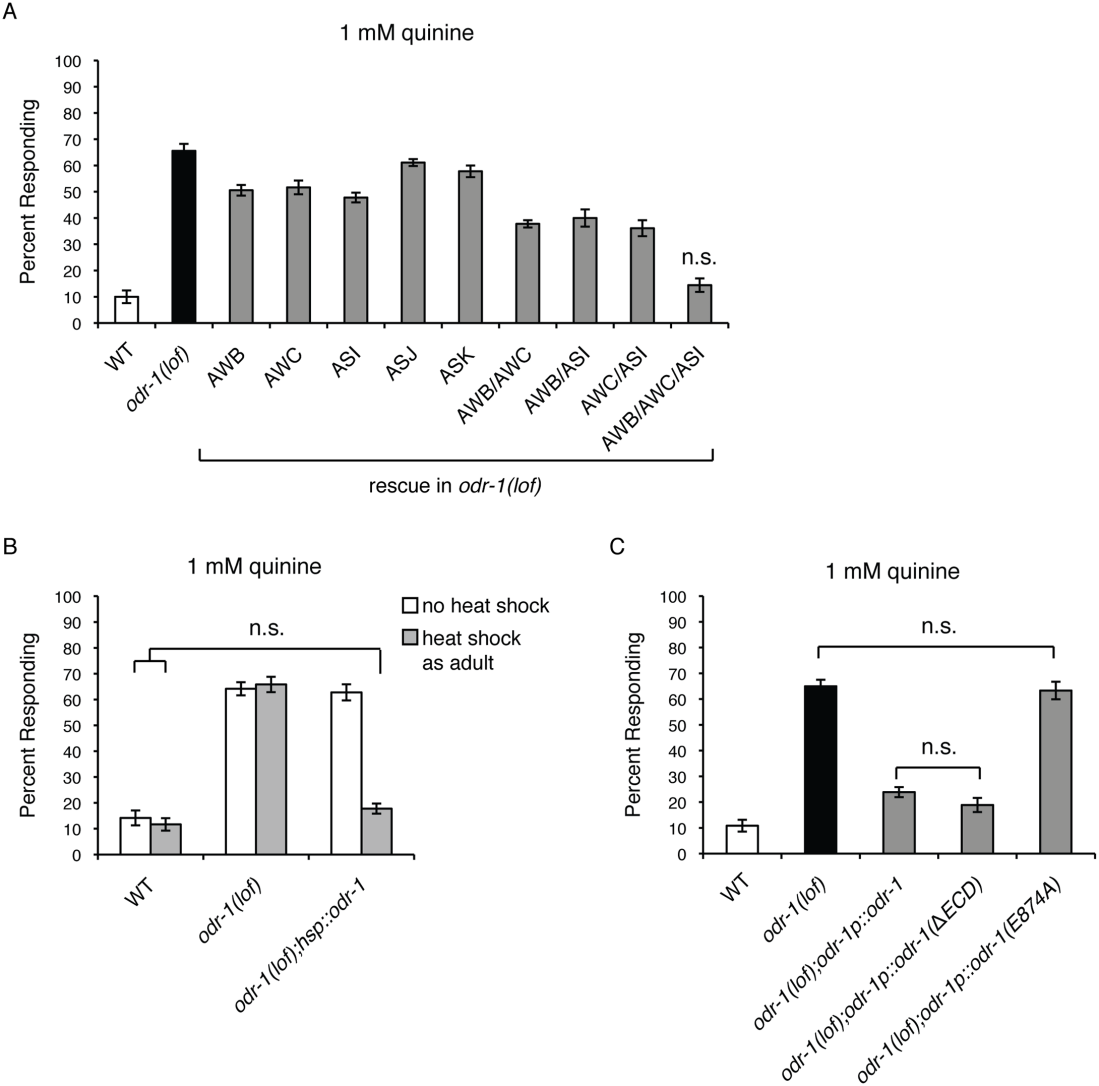
ODR-1 functions in adult sensory neurons. (A) ODR-1 expression in the AWB, AWC and ASI sensory neurons is sufficient to rescue the behavioral hypersensitivity of *odr-1(lof)* animals. The *str-1* [66], *ceh-36p3* [65], *gpa-4* [64], *trx-1* [63] and *srbc-66* [62] promoters were used to drive expression of wild-type *odr-1* (genomic sequence) in *odr-1(lof)* animals. These promoters drive expression in the following cells: *str-1* (AWB), *ceh-36p3* (AWC), *gpa-4* (ASI), *trx-1* (ASJ and intestinal cells), *srbc-66* (ASK). While more *odr-1(lof)* animal respond to 1 mM quinine than do wild-type animals, co-expressing *odr-1* in the AWB, AWC and ASI sensory neurons returned response to wild-type levels (p > 0.1). (B) ODR-1 functions in adult animals to regulate behavioral sensitivity. Adult *odr-1(lof)* animals expressing *odr-1* (genomic sequence) under the control of a heat shock inducible promoter (hsp) [68] were tested without heat shock (white bars) or 4 hours after heat shock treatment (grey bars). While *odr-1(lof)* animals have a hypersensitive response to dilute (1 mM) quinine, heat shock induced expression of *odr-1* in adult *odr-1(lof)* animals abolished this hypersensitivity and returned quinine response to the degree seen in wild-type animals (p > 0.1 when comparing *odr-1(lof)* animals with heat shock treatment to wild-type animals either with or without heat shock). (C) The extracellular domain of ODR-1 is not required for regulation of quinine sensitivity. In *odr-1(lof)* animals, the *odr-1* promoter was used to drive expression of either wild-type ODR-1 (genomic sequence), ODR-1 lacking its extracellular domain (ΔECD) or ODR-1 with a point mutation (E874A) that abolishes GTP binding in the catalytic domain. ODR-1(ΔECD) rescued the hypersensitivity of *odr-1(lof)* animals as well as wild-type ODR-1 (p > 0.1). Expression of ODR-1(E874A) had no effect on response sensitivity (p > 0.5 when compared to *odr-1(lof)* animals). The percentage of animals responding is shown. The combined data of ≥ 3 independent lines and n ≥ 120 transgenic animals is shown in each panel. Allele used: *odr-1(n1936)* loss-of-function. WT = the N2 wild-type strain. lof = loss-of-function. n.s. = not significant.

We previously reported that animals lacking the function of the guanylyl cyclase ODR-1 are hypersensitive in their behavioral avoidance response to dilute concentrations of the bitter tastant quinine [58]. Significantly more *odr-1* loss-of-function (lof) animals respond to dilute (1 mM) quinine than wild-type animals (Fig 1) [58]. The ASH sensory neurons are the main cells used to detect quinine in *C*. *elegans*, but the ASK neurons also contribute [52]. ODR-1 is not expressed in ASH, but is expressed in five other head sensory neurons—AWB, AWC, ASI, ASJ and ASK [60,61]. To determine in which cell(s) ODR-1 function is sufficient to dampen quinine sensitivity, we restored ODR-1 function in each neuron pair that it is natively expressed in, using the following cell-specific or -selective promoters: *str-1p* (AWB), *ceh-36p3* (AWC), *gpa-4p* (ASI), *trx-1p* (ASJ) and *srbc-66p* (ASK) [62–66]. While expressing ODR-1 in ASJ or ASK had only a minimal effect on quinine sensitivity, individually expressing ODR-1 in either the AWB, AWC or ASI sensory neurons partially rescued the *odr-1(lof)* quinine hypersensitivity phenotype (Fig 1A). This suggested that ODR-1 function in more than one neuron pair regulates the quinine response. Co-injection of *str-1p*::*odr-1*, *ceh36p3*::*odr-1* and *gpa-4p*::*odr-1* to simultaneously express ODR-1 in the AWB, AWC and ASI sensory neurons of *odr-1(lof)* animals returned quinine response to the level seen when *odr-1* was expressed under the control of its own promoter (*odr-1p*::*odr-1*), consistent with ODR-1 functioning in multiple neurons to regulate quinine sensitivity (Fig 1).

### ODR-1 Function in Adult Animals Is Sufficient to Regulate Behavioral Sensitivity to Quinine

*odr-1(lof)* animals develop with altered membraneous structures at the distal segments of the AWB, but not ASI, dendritic cilia [67]; AWC cilia structure was not examined in this study. To assess when ODR-1 function is required to downregulate quinine sensitivity, the *odr-1* gene was placed under the control of a heat shock inducible promoter [68] and introduced into *odr-1(lof)* animals. Induction of *odr-1* expression by heat shock in adult animal stages returned the behavioral response to dilute quinine to wild-type levels when assayed four hours later (Fig 1B). Transgenic animals that were not heat shocked remained hypersensitive, similar to *odr-1(lof)* animals (Fig 1B). These results demonstrate that, even though cilia morphogenesis is likely an active process that continues through the late larval stages [67], *odr-1* is only required in adult stages for normal behavioral sensitivity to dilute quinine. This heat shock-induced expression of *odr-1* is also after developmental cell fate specification and neuronal connectivity is complete.

### The Extracellular Domain of ODR-1 Is Not Required for Regulation of Quinine Sensitivity

ODR-1 is a receptor-type guanylyl cyclase with an extracellular domain (ECD) and an intracellular catalytic domain that processes GTP into cGMP [61]. To assess the contribution of the ECD to ODR-1 function in the regulation of quinine avoidance behavioral sensitivity, we expressed an ODR-1 construct lacking the extracellular domain (ΔECD) [61] under the control of its native promoter in *odr-1(lof)* animals (Fig 1C). Expression of the *odr-1p*::*odr-1(ΔECD)* construct restored quinine sensitivity to wild-type levels, similar to the expression of the wild-type *odr-1* construct (*odr-1p*::*odr-1*) (Fig 1C). This suggests that the extracellular receptor region is not necessary for ODR-1 function in modulating quinine sensitivity.

To determine whether ODR-1’s ability to produce cGMP is necessary for regulation of quinine sensitivity, we expressed ODR-1 harboring a point mutation that abolishes GTP binding in the catalytic domain [61]. *odr-1(lof)* animals expressing *odr-1p*::*odr-1(E874A)* remained hypersensitive in their response to quinine (Fig 1C). This indicates an important role for cGMP in modulation of the quinine response and is consistent with our previous demonstration that the *C*. *elegans* cGMP-dependent protein kinase EGL-4 also regulates quinine response sensitivity [58].

### The AWB, AWC and ASI Sensory Neurons Dampen Quinine Sensitivity

**Fig 2:**
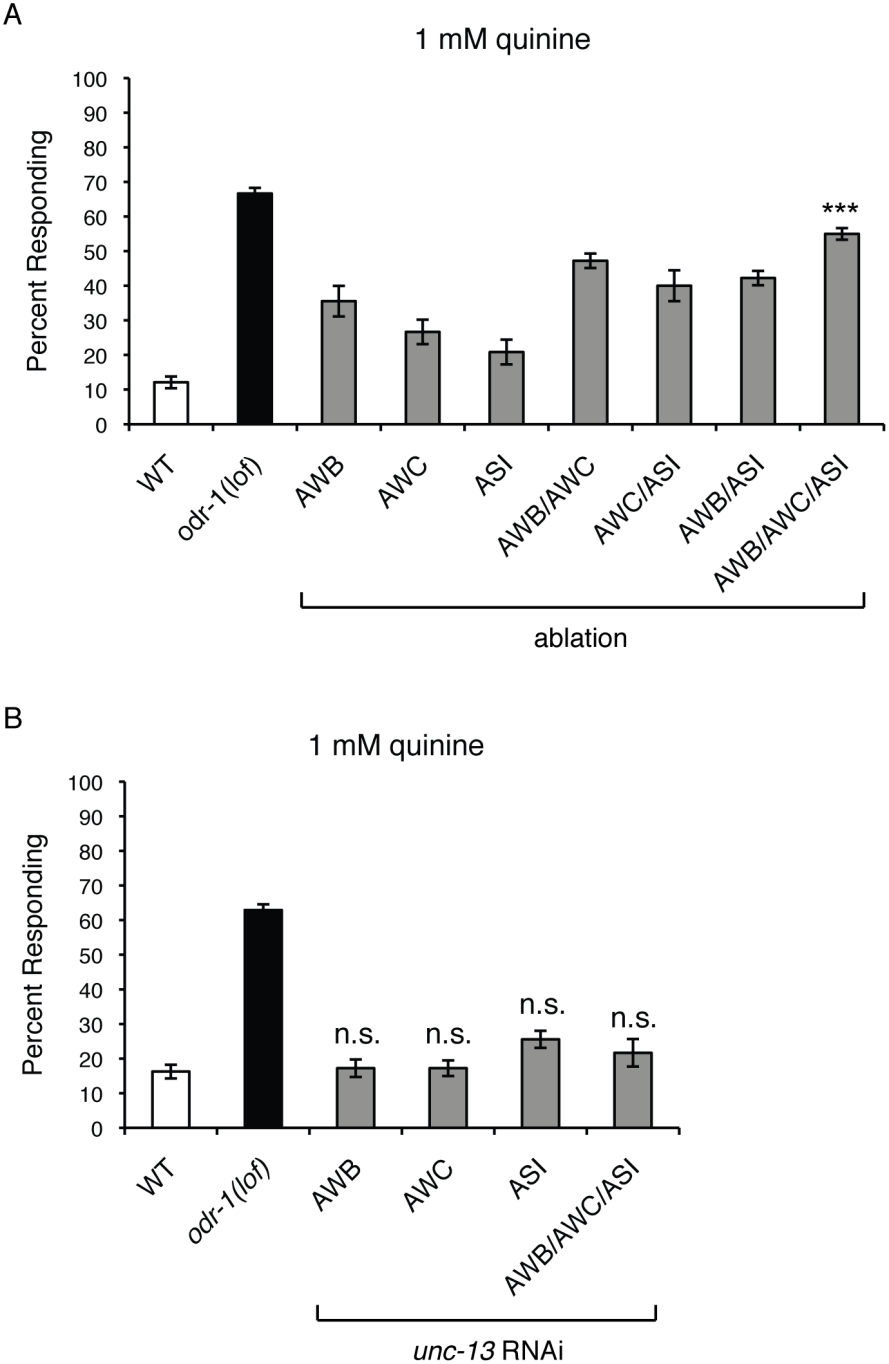
Neurotransmitter release from the AWB, AWC and ASI sensory neurons is not required to modulate quinine sensitivity. (A) Genetic ablation of the AWB, AWC and ASI sensory neurons resulted in quinine hypersensitivity. The reconstituted caspase approach [69] was used to genetically ablate AWB, AWC [70] and ASI [70] either individually or in combination in otherwise wild-type animals. In each case, some hypersensitivity was observed (p < 0.05 for each when compared to wild-type). When all three neuron pairs were ablated simultaneously, the largest degree of behavioral hypersensitivity to dilute (1 mM) quinine was observed (p < 0.0001). (B) UNC-13-dependent synaptic signaling from AWB, AWC and ASI does not modulate quinine sensitivity. The *str-1* (AWB) [66], *ceh-36p3* (AWC) [65] and *gpa-4* (ASI) [64] promoters were used to co-express a non-coding fragment of *unc-13* in both the sense and antisense orientations of otherwise wild-type animals. RNAi knock-down of *unc-13* in the AWB, AWC or ASI neurons, either individually or in combination, did not result in behavioral hypersensitivity to dilute (1 mM) quinine (p > 0.05 when compared to WT animals for all transgenes). The percentage of animals responding is shown. The combined data of ≥ 3 independent lines and n ≥ 120 transgenic animals is shown in each panel. Alleles used: *odr-1(n1936)* loss-of-function. WT = the N2 wild-type strain. lof = loss-of-function. n.s. = not significant. See also S1 Fig.

The above results suggested a modulatory role for the AWB, AWC and ASI neurons in quinine behavioral sensitivity. To further examine their contribution to the regulation of the quinine response, we genetically ablated these cells in wild-type animals, both as individual neuron pairs and in combination, using a reconstituted caspase approach [69,70]. Ablation of either the AWBs, AWCs or ASIs did not produce a marked hypersensitive phenotype (Fig 2A). While animals lacking two of the three neuron pairs displayed greater hypersensitivity than the single ablates, ablation of all three neuron pairs together resulted in the greatest degree of quinine hypersensitivity (Fig 2A). Taken together, our results reveal a role for the AWB, AWC and ASI sensory neurons in the negative regulation of quinine avoidance, and further suggest that ODR-1 function in these cells contributes to their modulatory role.

### Neurotransmitter Release from AWB, AWC and ASI Is Not Required for the Modulation of Quinine Sensitivity

As the ASH nociceptors are the primary cells used to detect quinine [52], we sought to determine how the AWB, AWC and ASI sensory neurons might influence signaling through the ASH sensory circuit. One possibility could be via synaptic signaling between these neurons. UNC-13 protein is required for synaptic vesicle fusion and neurotransmitter release at synapses [71,72], and AWB and ASI are known to form direct synaptic connections onto ASH [2]. Because *unc-13* null mutations are lethal, we used cell-specific RNAi [73] to knockdown *unc-13* in AWB, AWC and ASI and block synaptic transmission from these neurons. We confirmed efficient *unc-13* RNAi knockdown using chemosensory assays that require synaptic signaling from AWB and AWC for proper behavioral responses (S1 Fig). Animals in which *unc-13* was simultaneously knocked down in the AWB, AWC and ASI sensory neurons did not show increased sensitivity to dilute quinine (Fig 2B), suggesting that vesicular synaptic transduction is not the mechanism by which these neurons influence the ASH-mediated response to quinine.

### The INX-4 Innexin Gap Junction Component Is Required for Regulation of Quinine Sensitivity

**Fig 3:**
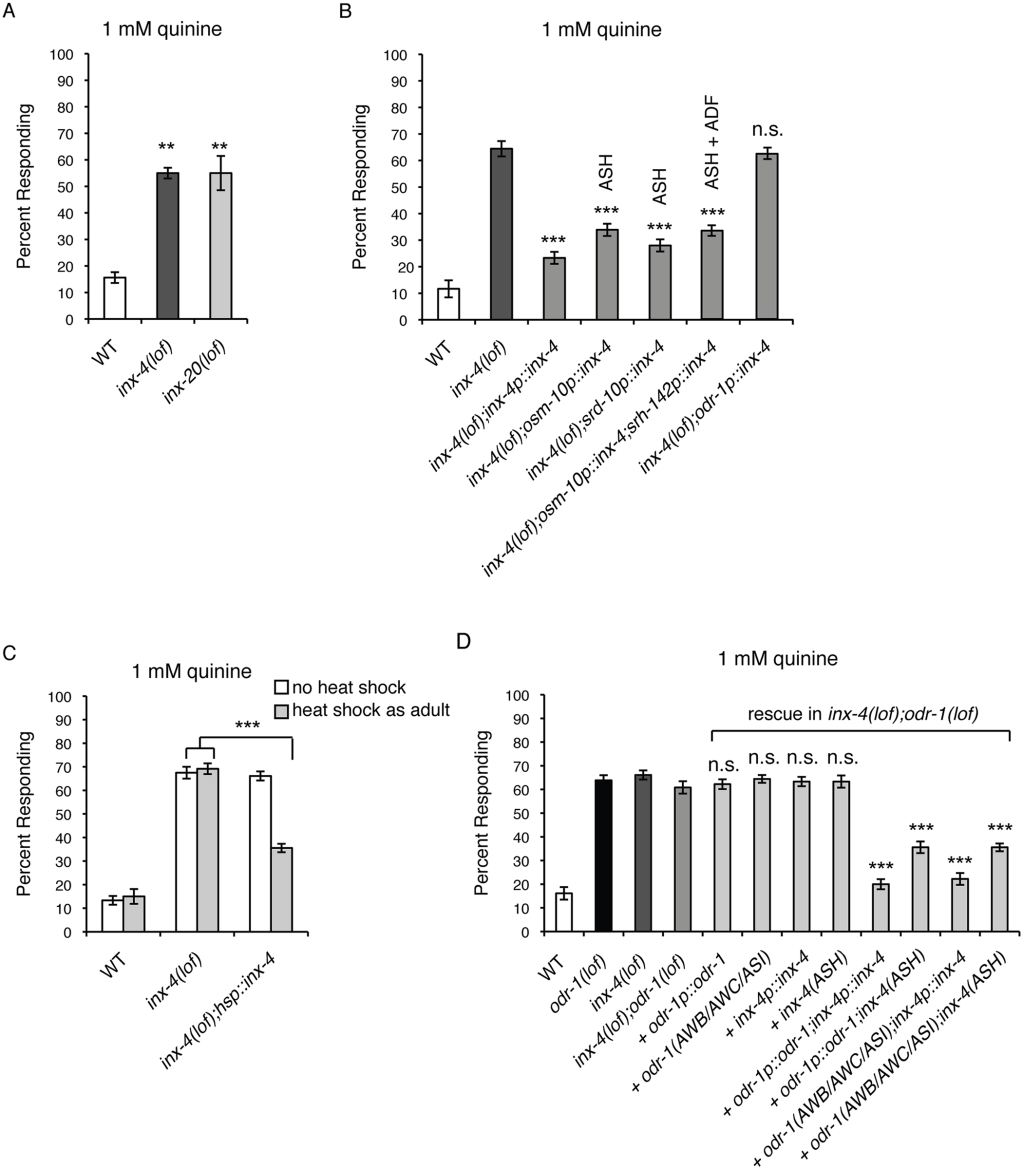
Gap junctions regulate quinine sensitivity. (A) Loss-of-function mutations in the innexin gap junction genes *inx-4* and *inx-20* resulted in behavioral hypersensitivity to 1 mM quinine (p < 0.001 for each when compared to wild-type animals). (B) The *inx-4* [35], *osm-10* (ASH, ASI, PHA and PHB) [48], *srd-10* (ASH, PHA and PHB), *srh-142* (ADF) [79] and *odr-1* promoters were used to drive expression of wild-type *inx-4* cDNA in *inx-4(lof)* animals. ASH-selective expression significantly dampened the *inx-4(lof)* hypersensitive response (p < 0.0001), while expression in the ODR-1-expressing neurons had no effect (p > 0.4). (C) INX-4 functions in adult animals to regulate behavioral sensitivity. Adult *inx-4(lof)* animals expressing *inx-4* cDNA under the control of a heat shock inducible promoter (hsp) [68] were tested without heat shock (white bars) or 4 hours after heat shock treatment (grey bars). While *inx-4(lof)* animals had a hypersensitive response to dilute (1 mM) quinine, heat shock induced expression of *inx-4* in adult *inx-4(lof)* animals dampened this hypersensitivity (p < 0.0001 when comparing *inx-4(lof);hsp*::*inx-4* animals with heat shock treatment to *inx-4(lof)* animals either with or without heat shock). (D) INX-4 gap junctions are required for ODR-1 modulation of quinine sensitivity. Expression of only *odr-1* or *inx-4* in *odr-1(lof);inx-4(lof)* animals had no effect on quinine hypersensitivity (p > 0.2 when comparing rescue of either to *odr-1(lof);inx-4(lof)* animals). Simultaneous expression of *inx-4* in ASH using the *osm-10p* promoter [48] and expression of *odr-1* either behind its native promoter or using AWB, AWC and ASI cell-selective promoters (*str-1* [66], *ceh-36p3* [65], and *gpa-4* [64], respectively), rescued hypersensitivity (p < 0.0001). The percentage of animals responding is shown. The combined data of ≥ 3 independent lines and n ≥ 120 transgenic animals is shown in each panel. Alleles used: *inx-4(ok2373)* loss-of-function, *inx-20(ok426)* loss-of-function and *odr-1(n1936)* loss-of-function. WT = the N2 wild-type strain. lof = loss-of-function. n.s. = not significant. See also S2 and S3 Figs.

A second way in which neurons can communicate with each other is via diffusion of ions and small metabolites through gap junctions. Studies in mammalian systems have suggested that cyclic nucleotides can also pass through gap junctions to affect cellular function. For example, cAMP movement between cells has been visualized following ectopic expression of gap junction components in human tissue culture [29,74,75]. cAMP movement through gap junctions also suppresses CD4+ T-cell function [76] and may alter gene expression in myelinating Schwann cells [77]. In addition, cGMP can pass through gap junctions in human cell culture, as well as between cultured mouse follicle cells and oocytes [29,30,32,78]. While 25 innexins are encoded by the *C*. *elegans* genome, the physiological roles of most are unknown [35–37]. To determine whether gap junction signaling can modulate quinine sensitivity, we assayed animals with loss-of-function alleles for 16 of the 25 innexins encoded by the *C*. *elegans* genome for response to dilute (1 mM) quinine (see Supplemental Materials and Methods). Two innexin mutants, *inx-4(lof)* and *inx-20(lof)*, responded better than wild-type animals to dilute quinine (Fig 3A). While *inx-20* expression has only been reported in the pharyngeal epithelium and the pm2 pharyngeal muscle cell, *inx-4* expression was seen in several head and tail neurons, including the ASHs of early larvae and the ADFs of L1s [35]. Using an *inx-4p*::*gfp* reporter construct, we have also confirmed *inx-4* expression in the ASH nociceptors of adult animals (S2 Fig).

To determine whether INX-4 function in the ASHs is sufficient to regulate quinine response, the ASH cell-selective promoters *osm-10* [48] and *srd-10* were used to restore INX-4 function and animals were assayed for response to 1 mM quinine. Expression in ASH using either promoter dampened the *inx-4(lof)* hypersensitive response, while simultaneous expression of INX-4 in ASH and ADF, using the *osm-10* [48] and *srh-142* [79] promoters, respectively, did not result in additional rescue (Fig 3B). Furthermore, consistent with the reported lack of *inx-4* expression in ODR-1-expressing neurons [35], an *odr-1p*::*inx-4* construct did not rescue the quinine hypersensitivity of *inx-4(lof)* animals (Fig 3B). These results suggest that the ASH nociceptors are the primary site for INX-4 function in regulating quinine sensitivity.

Since *inx-4* is expressed from larval through adult stages, the *inx-4* gene was placed under the control of a heat shock inducible promoter [68] and introduced into *inx-4(lof)* animals to determine when *inx-4* function is required to modulate quinine sensitivity. Induction of *inx-4* expression by heat shock in adult animal stages significantly dampened the behavioral hypersensitivity of *inx-4(lof)* animals to dilute quinine when assayed four hours later (Fig 3C). Transgenic animals that were not heat shocked remained hypersensitive, similar to *inx-4(lof)* animals (Fig 3C). These results demonstrate that, like ODR-1 (Fig 1B), INX-4 function in adult animal stages is sufficient to modulate behavioral sensitivity to dilute quinine.

### ODR-1 and INX-4 Do Not Regulate ASH Sensitivity in General

We previously found that *egl-4(lof)* animals are hypersensitive in their response to distinct ASH-detected stimuli and, although EGL-4 functions in the ASH sensory neurons to regulate these behaviors, this cGMP-dependent protein kinase does not regulate ASH sensitivity in general [58]. For example, *egl-4(lof)* animals respond normally to the bitter tastant primaquine, the detergent SDS and the heavy metal copper [58], all of which are detected by the ASH nociceptors [49,51–53]. To determine whether loss of *odr-1* or *inx-4* increased overall sensitivity of ASH, or also selectively affected sensory signaling, we assayed *odr-1(lof)* and *inx-4(lof)* animals for their response to primaquine, SDS and copper. For each of these stimuli, *odr-1(lof)* and *inx-4(lof)* animals responded similarly to wild-type animals (S3 Fig), indicating that ODR-1 and INX-4 also do not regulate ASH signaling in general.

### INX-4 Gap Junctions Are Required for ODR-1-Mediated Downregulation of Quinine Sensitivity

If INX-4 functions in the same pathway as ODR-1 to dampen quinine sensitivity, then *odr-1(lof);inx-4(lof)* double mutant animals should display a behavioral phenotype similar to *odr-1(lof)* animals and the hypersensitivity should not be additive. We found that the double mutant animals’ response to dilute (1 mM) quinine was indistinguishable from animals lacking only ODR-1 function (p > 0.2, Fig 3D), suggesting that they do function in the same regulatory pathway. Consistent with this observation, in the *odr-1(lof);inx-4(lof)* background, expression of only either *odr-1* or *inx-4* had no effect on the quinine hypersensitivity (Fig 3D). However, simultaneous expression of *inx-4* in ASH and expression of *odr-1* either behind its native promoter or using AWB, AWC and ASI cell-selective promoters, rescued hypersensitivity (Fig 3D).

### cGMP Generation in ASH Is Sufficient to Dampen Behavioral Sensitivity to Quinine

**Fig 4:**
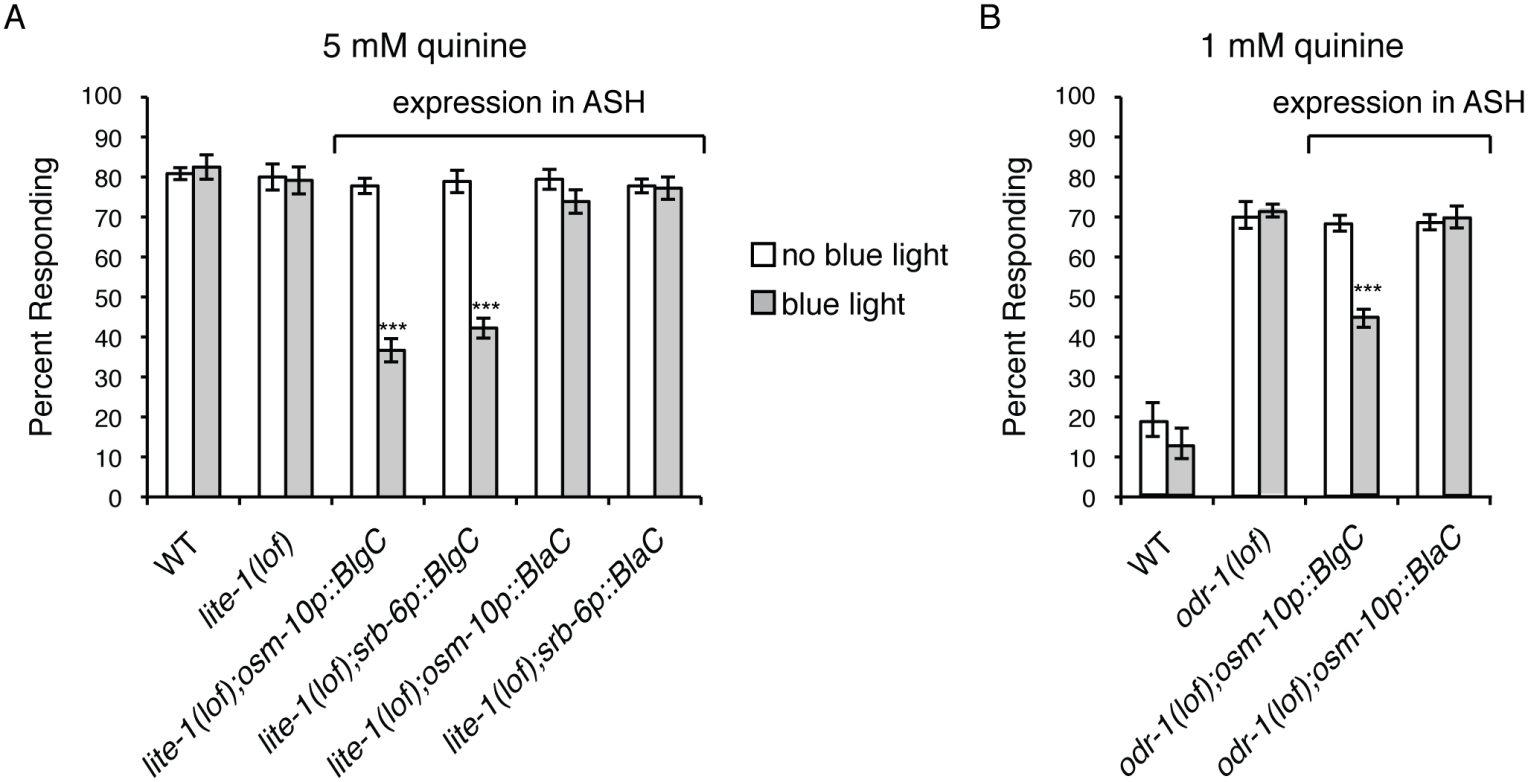
cGMP generation in ASH dampens quinine sensitivity. The ASH-selective *osm-10* [48] and *srb-6* [80] promoters were used to drive expression of a blue light-inducible guanylyl cyclase (BlgC) or blue light-inducible adenylyl cyclase (BlaC) [81]. *osm-10p* drives expression in ASH and weakly in ASI in the head, and in PHA and PHB in the tail. *srb-6p* drives expression in the ASH, ADL and ADF head sensory neurons, and in PHA and PHB in the tail. Adult animals expressing BlgC or BlaC were tested without blue light exposure (white bars) or after a 30 second exposure (grey bars). (A) While *lite-1(lof)* animals responded robustly to 5 mM quinine, similar to wild-type animals (p > 0.4), transgenic animals expressing BlgC displayed a significantly diminished response following blue light exposure (p < 0.0001 for each ASH-selective promoter). Transgenic animals expressing BlaC remained sensitive following blue light exposure (p > 0.05 when compared to wild-type animals). (B) While *odr-1(lof)* animals were hypersensitive in their response to 1 mM quinine, transgenic animals expressing BlgC displayed a significantly diminished response following blue light exposure (p < 0.0001). Transgenic animals expressing BlaC remained hypersensitive following blue light exposure (p > 0.5 when compared to *odr-1(lof)* animals). The percentage of animals responding is shown. The combined data of ≥ 2 independent lines and n ≥ 95 transgenic animals is shown. Alleles used: *lite-1(ce314)* loss-of-function, *odr-1(n1936)* loss-of-function. WT = the N2 wild-type strain. lof = loss-of-function. See also S4 Fig.

No guanylyl cyclase has been found to be expressed in ASH [58,59] and the results described above suggest that cGMP generated at distant sites can dampen ASH response to sensory stimuli. To determine if the mere presence of cGMP in ASH is sufficient to dampen quinine sensitivity, the ASH cell-selective promoters *osm-10* [48] and *srb-6* [80] were used to express a blue light-inducible guanylyl cyclase (BlgC) [81] in the ASHs of animals lacking the blue-violet light receptor LITE-1 [82]. When assayed 10 minutes after a 30-second exposure to blue light, animals expressing BlgC in the ASH sensory neurons displayed a diminished response to both 5 mM (Fig 4A) and 10 mM quinine (S4 Fig), while transgenic animals that were not flashed with blue light displayed wild-type sensitivity. Although cAMP generation by BlgC was undetectable in *E*. *coli*, BlgC was shown to posses ~10% residual adenylyl cyclase activity *in vitro* [81]. To confirm that the dampened quinine response was due to production of cGMP, and not cAMP, we also assayed animals expressing a blue light-inducible adenylyl cyclase, BlaC [81]. Unlike BlgC, blue light-induction of BlaC to stimulate cAMP production did not alter animals’ response to quinine (Fig 4A and S4 Fig). Blue light induction of BlgC in the ASHs was also sufficient to significantly diminish the hypersensitivity of *odr-1(lof)* animals in response to 1 mM quinine (Fig 4B). Together, these results demonstrate that elevating cGMP levels in ASH is sufficient to dampen behavioral sensitivity to quinine.

### The ADF, AFD and AIA Neurons That Form Gap Junctions with ASH Modulate Quinine Sensitivity

**Fig 5:**
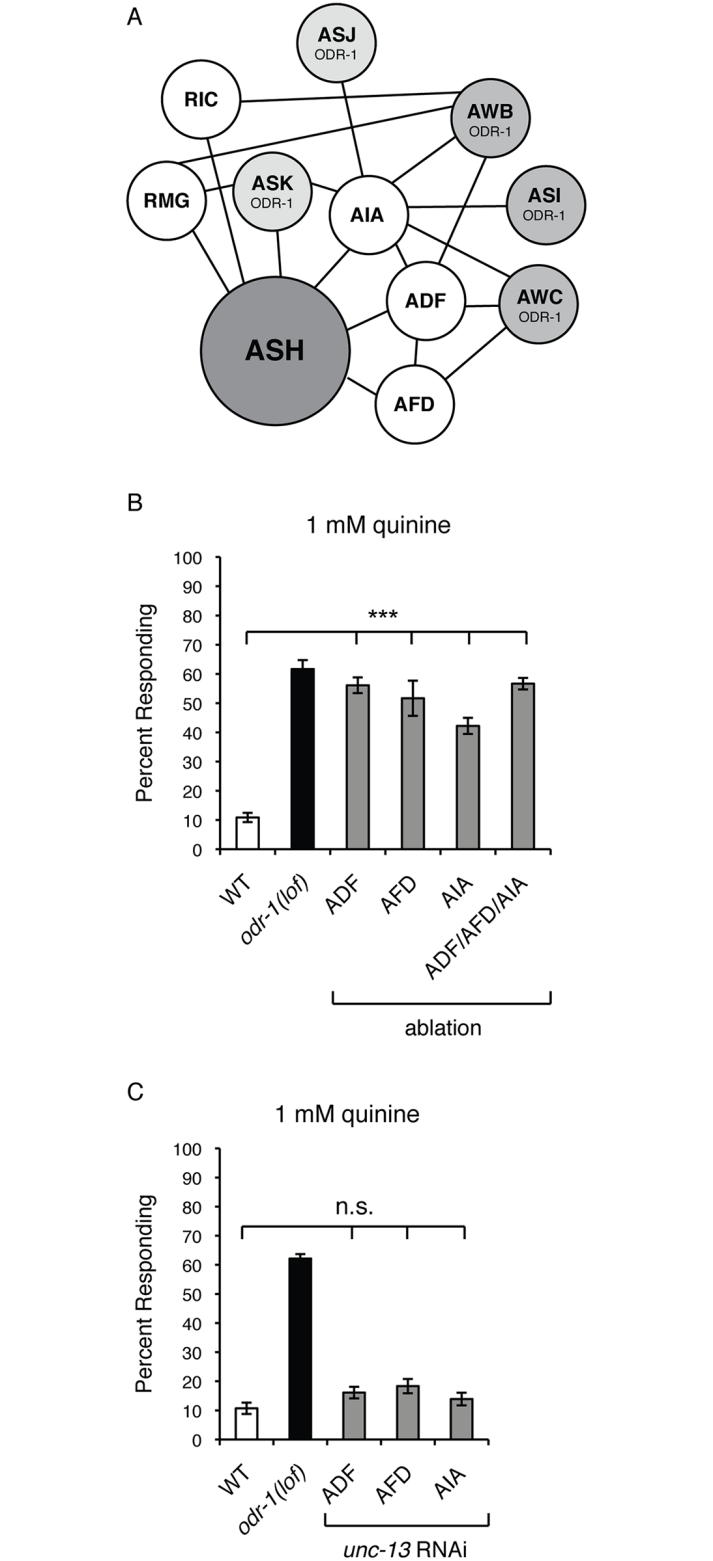
ADF, AFD and AIA neurons that form gap junctions with ASH modulate quinine sensitivity. (A) A diagram of the gap junction network linking the AWB, AWC and ASI sensory neurons to ASH is shown. This diagram reflects the most current wiring annotations of the original electron micrograph series reported in [2], as curated at WormWiring.org. Black lines indicate gap junction connections between neurons. Only those neurons and gap junctions that would allow the ODR-1-expressing neurons to ultimately connect to ASH are shown. The head sensory neurons that express ODR-1 are shown. The ODR-1-expressing sensory neurons AWB, AWC and ASI do not form gap junction connections directly with ASH. However, these neurons are connected to ASH indirectly through a gap junction network via ADF (AWB, AWC), AFD (AWC), and AIA (AWC, AWB and ASI) [2]. (B) Genetic ablation of the ADF, AFD and AIA neurons resulted in quinine hypersensitivity. The reconstituted caspase approach [69] was used to genetically ablate ADF, AFD and AIA either individually or in combination in otherwise wild-type animals. Loss of any of the three neuron pairs individually, or all three neuron pairs simultaneously, resulted in significant behavioral hypersensitivity to dilute (1 mM) quinine (p < 0.0001 for each when compared to wild-type animals). (C) UNC-13-dependent synaptic signaling from ADF, AFD and AIA does not modulate quinine sensitivity. The *srh-142p* (ADF) [79], *gcy-8p* (AFD) [121] and *gcy-28dp* (AIA) [122] promoters were used to co-express a non-coding fragment of *unc-13* in both the sense and antisense orientations in otherwise wild-type animals. RNAi knock-down of *unc-13* in the ADF, AFD or AIA neurons did not result in behavioral hypersensitivity to dilute (1 mM) quinine (p > 0.05 when compared to wild-type animals for each transgene). The percentage of animals responding is shown. The combined data of ≥ 3 independent lines and n ≥ 120 transgenic animals is shown in each panel. Allele used: *odr-1(n1936)* loss-of-function. WT = the N2 wild-type strain. lof = loss-of-function. n.s. = not significant.

Based on the *C*. *elegans* hermaphrodite wiring diagram (we referred to WormWiring.org for the most current wiring annotations based on the original electron micrograph series reported in [2]; Scott Emmons, personal communication), the ODR-1-expressing sensory neurons AWB, AWC and ASI do not form gap junction connections directly with ASH. However, these neurons are connected to ASH indirectly through a gap junction network via ADF (AWB, AWC), AFD (AWC) and AIA (AWB, AWC and ASI) (Fig 5A). The ADFs are chemosensory, the AFDs are thermosensory neurons, and the AIAs are interneurons [43,83]. ADF is also directly connected to both AFD and AIA by gap junctions (Fig 5A). These connections reveal a neuronal circuitry wherein ADF, AFD and AIA lay between the cGMP generating neurons (AWB, AWC and ASI) and ASH. We note that RMG and RIC also connect AWB to ASH, but their role in modulating the quinine response was not examined in this study. To determine whether ADF, AFD or AIA regulate the quinine response, we genetically ablated these cells in wild-type animals, both as individual neuron pairs and in combination, again using reconstituted caspases [69,84]. As shown in Fig 5B, loss of any one of the three neuron pairs resulted in significant behavioral hypersensitivity, as did simultaneous ablation of all three. In fact, the degree of quinine hypersensitivity seen in the ADF/AFD/AIA ablated animals was indistinguishable from that of the AWB/AWC/ASI animals (p > 0.5).

We noted that ADF and AIA do also chemically synapse onto ASH [2] (and WormWiring.org). To confirm that vesicular synaptic signaling from these neurons does not underlie their ability to decrease ASH sensitivity, we again utilized cell-specific RNAi [73] to knockdown *unc-13* in ADF and AIA to block synaptic transmission from these neurons. Animals in which *unc-13* was knocked down in either the ADF or AIA neurons did not show increased sensitivity to dilute quinine (Fig 5C). Even though AFD does not synapse onto ASH, we also confirmed that *unc-13* RNAi in this neuron pair did not affect quinine sensitivity (Fig 5C). Taken together, our data suggest that gap junction-mediated communication between AWB/AWC/ASI and ASH, via ADF/AFD/AIA can regulate ASH sensitivity and an animal’s response to environmental stimuli.

**Fig 6:**
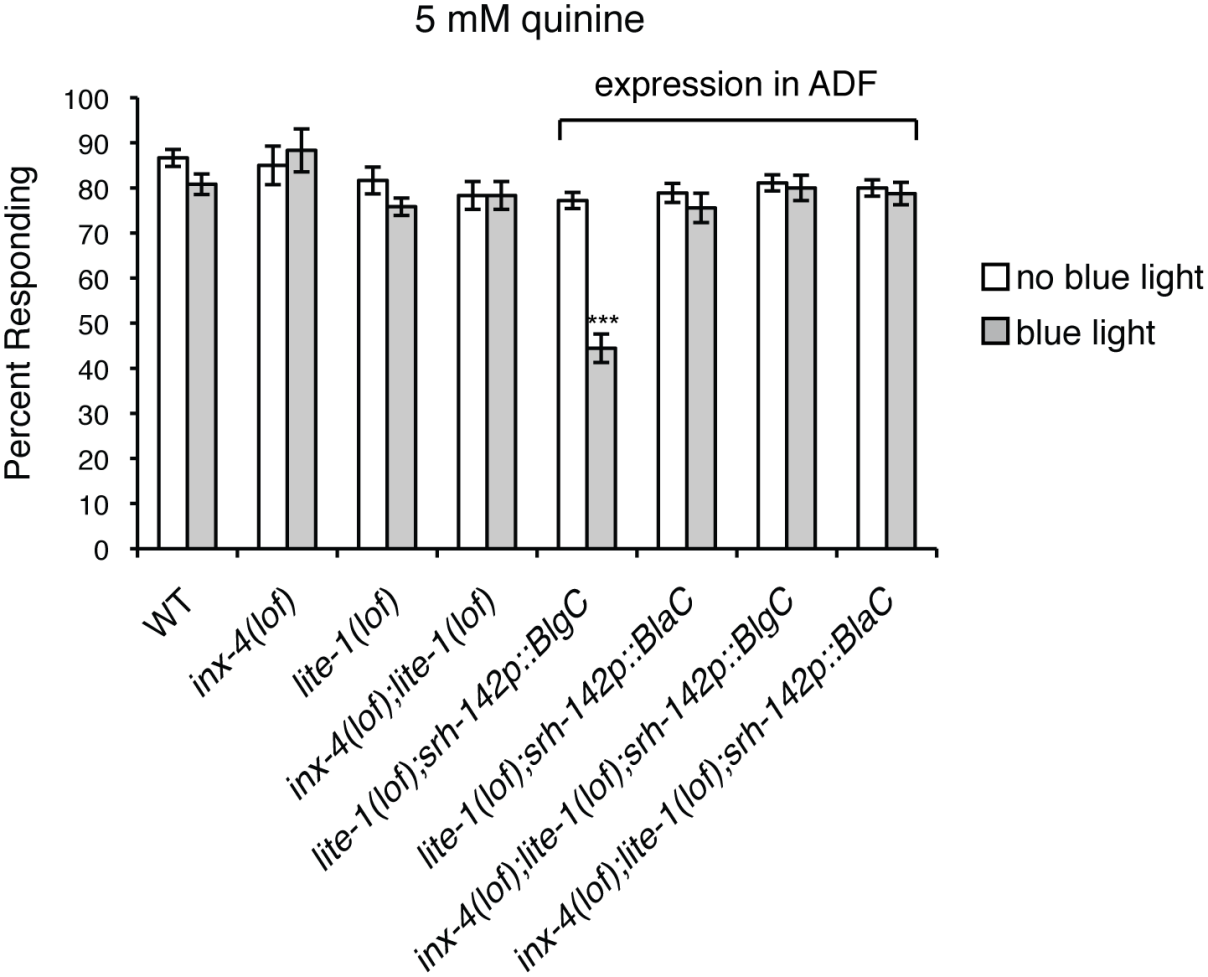
cGMP generation in ADF dampens quinine sensitivity. The ADF-specific *srh-142* promoter [79] was used to drive expression of a blue light-inducible guanylyl cyclase (BlgC) or blue light-inducible adenylyl cyclase (BlaC) [81] in *lite-1(lof)* or *inx-4(lof);lite-1(lof)* animals. *srh-142p* drives expression only in the two ADF head sensory neurons. Adult *lite-1(lof)* or *inx-4(lof);lite-1(lof)* animals expressing BlgC or BlaC were tested without blue light exposure (white bars) or after a 30 second exposure and 30 second recovery (grey bars). While *lite-1(lof)* animals responded robustly to 5 mM quinine, similar to WT animals (p > 0.1), transgenic animals expressing BlgC in the ADFs displayed a significantly diminished response following blue light exposure (p < 0.0001). Transgenic animals expressing BlaC did not display a diminished response following blue light exposure (p > 0.2 when compared to wild-type animals). *inx-4(lof);lite-1(lof)* transgenic animals expressing either BlgC or BlaC did not display a diminished response following blue light exposure (p > 0.7 when compared to wild-type animals). The percentage of animals responding is shown. The combined data of ≥ 3 independent lines and n ≥ 120 transgenic animals is shown in each panel. Alleles used: *lite-1(ce314)* loss-of-function and *inx-4(ok2373)* loss-of-function. WT = the N2 wild-type strain. lof = loss-of-function. See also S5 Fig.

No guanylyl cyclases are known to be expressed in the ADF sensory neurons [59]. Therefore, we next sought to determine whether ectopic cGMP generation in these cells, which lay between the ODR-1*-*expressing neurons and the ASHs in the gap junction network (Fig 5A), would be sufficient to dampen quinine sensitivity. The ADF-specific *srh-142* promoter [79] was used to drive expression of BlgC [81] in *lite-1(lof)* or *inx-4(lof);lite-1(lof)* animals. After exposure to blue light, *lite-1(lof)* animals expressing BlgC in the ADFs displayed a diminished response to 5 mM (Fig 6) and 10 mM (S5 Fig) quine, while transgenic animals that were not exposed to blue light displayed wild-type sensitivity. Conversely, *inx-4(lof);lite-1(lof)* transgenic animals expressing BlgC in the ADFs did not display a diminished response following blue light exposure (Fig 6 and S5 Fig). Consistent with the results described above (Fig 4A), blue light-induction of BlaC to stimulate cAMP production in the ADFs did not alter animals’ response to quinine (Fig 6 and S5 Fig). Together, these data demonstrate that elevating cGMP levels in the ADFs is sufficient to dampen behavioral sensitivity to quinine, and that function of the INX-4 gap junction component is required for this effect.

## Discussion

The sheer number of possible circuit outcomes revealed by anatomical wiring diagrams means that we cannot possibly predict how information might flow through a circuit based on physical connections alone. Even in the relatively simple nervous system of *C*. *elegans*, the function of most of the connections, or which connections will be preferentially used under different circumstances, is not known [85]. For example, neuropeptide signaling regulates a sensory context-dependent switch in the composition of a *C*. *elegans* salt sensory circuit [86]. In this case, one of the AWC olfactory sensory neurons is recruited to function as an interneuron during response to high salt concentrations [86]. It was also recently reported that, in a reciprocal inhibition circuit that fine-tunes copper (a heavy metal) avoidance, the ADF sensory neurons can be activated by neuropeptides to act as interneurons downstream of the ASI sensory neurons. The ADFs then dampen ASH sensitivity via serotonin release [87]. In addition, the *C*. *elegans* nose touch circuit appears to utilize electrical synapses to mediate both excitatory and inhibitory interactions between neurons [88]. Adding another layer of complexity, the internal state of an animal, such as its nutritional status and degree of satiety, can influence its sensitivity to environmental stimuli [58,89–93], and may even alter which neurons participate in stimulus detection [89].

Sensory systems in particular may be subject to extensive modulation [8,15]. For example, feeding state and food availability can alter gustatory and olfactory responses in diverse species, where the complexity of the nervous systems ranges from just hundreds of neurons to billions of neurons. To better understand the neuromodulatory mechanisms that regulate chemosensation, we focused on one component of the regulatory pathway that controls *C*. *elegans* response to aversive sensory stimuli, the guanylyl cyclase ODR-1. As previously reported, the two AWC olfactory neurons require ODR-1 function to mediate chemotaxis towards attractive odorants that they detect, while the AWBs require ODR-1 for 2-nonanone avoidance [61]. In addition, ODR-1 plays a role within the AWCs to regulate adaption in response to prolonged odor exposure [61]. Here, we describe a new role for ODR-1 in modulating animals’ avoidance response to the bitter tastant quinine in a non-cell-autonomous manner. This is, to our knowledge, the first *in vivo* demonstration of a guanylyl cyclase functioning to modulate the activity of another cell.

*odr-1(lof)* animals are hypersensitive in their response to dilute concentrations of the bitter tastant quinine, and ODR-1 function in three distinct pairs of sensory neurons (the AWBs, AWCs and ASIs) appears to contribute to the modulation of ASH-mediated avoidance (Fig 1). However, the evolutionary advantage of this sort of decentralized regulation of sensory signaling is not immediately clear. One possibility could be to prevent overstimulation of ASH, which is the main nociceptor in *C*. *elegans*. Nociceptors in general have high thresholds of activation, understandably to prevent organisms from unnecessarily reacting to minute or low-risk stimuli [94]. An additive modulatory role for AWB, AWC and ASI may help to assure that the threshold of ASH sensitivity to noxious stimuli remains high, consistent with the observed behavioral hypersensitivity we observed upon ablation of these three neurons pairs (Fig 2).

Another potential benefit of decentralized ASH regulation is that by utilizing multiple sensory neurons to regulate ASH function, the integration of diverse sets of environmental information can maximize the appropriateness of an animal’s response to its surroundings. For example, in addition to detecting the aversive odorants 100% octanol and 2-nonanone [66,89], which may indicate the presence of fungi or pathogenic bacteria [95–97], AWB also mediates lawn avoidance upon encountering the pathogenic bacteria *Serratia marcescens* [97]. All of these roles for AWB could help *C*. *elegans* avoid environments that might be harmful to them. Conversely, scents detected by AWC (benzaldehyde, butanone, isoamyl alcohol, 2,3-pentanedione, and 2,4,5-trimethylthiazole) [98,99] are primarily produced in nature by plants (e.g. fruits, nuts and coffee) and microorganisms. Therefore, these natural odorants signal potential sources of nutritive bacteria and it has been shown that *C*. *elegans* will populate areas of decaying plant matter [100]. Finally, if developing larvae encounter harsh environmental conditions, including elevated population density and/or a limited food supply, they can enter dauer arrest at the second molt [101]. This transition is repressed by the ASI, ADF and ASG neurons, which detect the dauer pheromone that serves as an indicator of population density. A high density of *C*. *elegans* in a given area could signify low or dwindling food availability there, which could in turn result in poor nutritional status. Thus, the AWBs, AWCs and ASIs detect distinct sets of environmental stimuli that can all provide the animal with information about potential food quality and availability.

**Fig 7:**
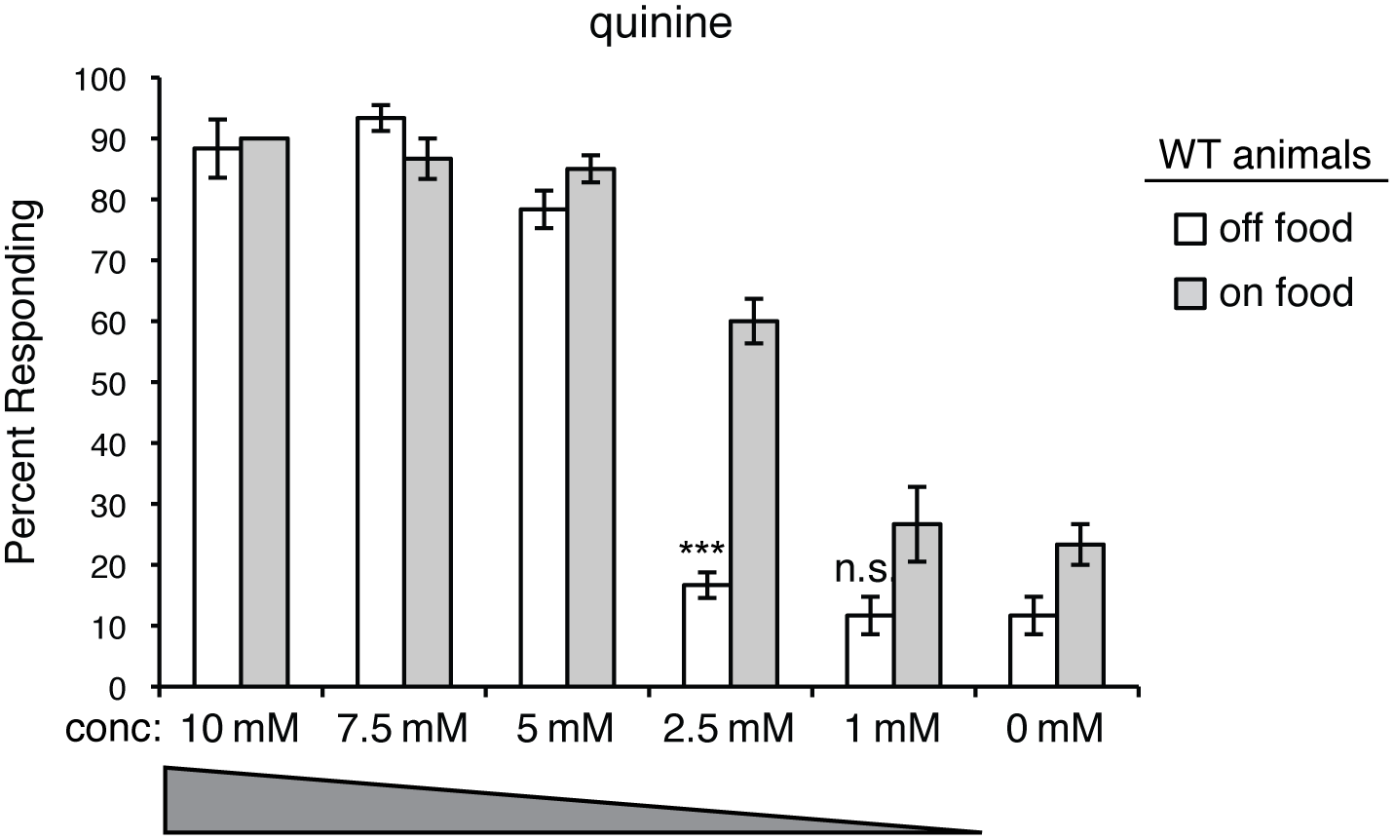
Response to quinine is modulated by feeding status. The response of wild-type animals to dilute quinine diminishes upon food deprivation (p < 0.00001 for 2.5 mM when comparing animals assayed “off food” versus “on food”). The percentage of animals responding is shown. The combined data of n ≥ 60 animals is shown for each concentration. Animals were assayed 10–20 minutes after transfer to plates with or without food (no bacterial lawn). conc = concentration. n.s. = not significant. WT = the N2 wild-type strain.

Following the initial detection of an environmental stimulus, the AWB, AWC or ASI sensory neurons may then subsequently modulate ASH sensitivity to indirectly optimize nociceptive responses in a context-dependent manner. For example, when animals are well-fed, they are more sensitive to aversive stimuli, including quinine (Fig 7), than they are upon food deprivation [58,89–93]. This may reflect the need of animals to reprioritize their behaviors to balance the need to avoid potentially dangerous situations with the need to find food. If starving, minimized aversive responses may maximize entry into new environments to increase the likelihood of encountering new food sources.

**Fig 8:**
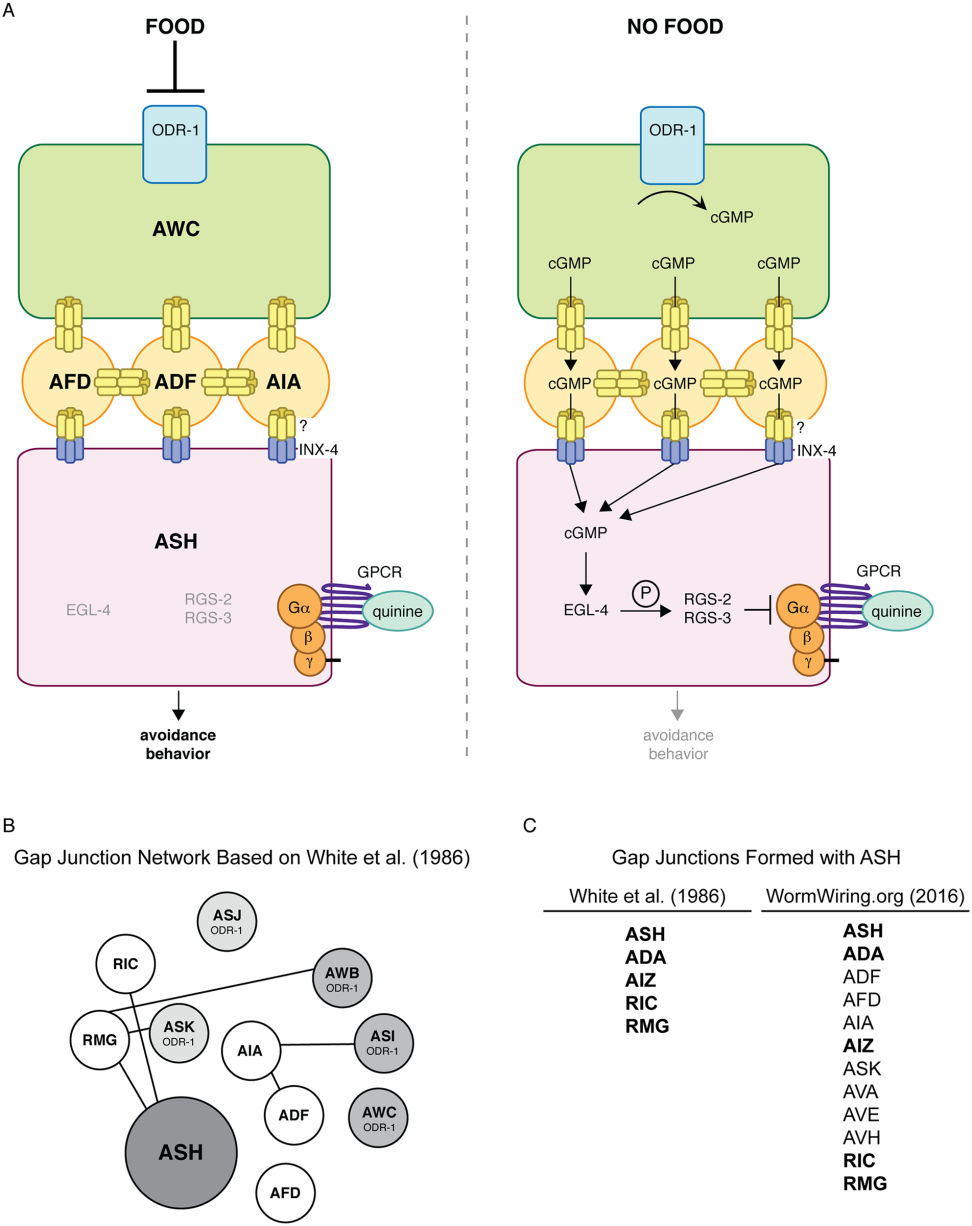
Model for ODR-1 modulation of ASH-mediated nociceptive signaling. (A) Our working model is that the transmembrane guanylyl cyclase ODR-1 functions in the AWB, AWC and ASI sensory neurons to decrease *C*. *elegans* behavioral sensitivity to the bitter tastant quinine (and possibly the volatile odorant octanol). Removal of food leads to cGMP accumulation, at least in the AWCs, likely by direct/indirect activation of ODR-1 in these neurons. Based on the re-annotated wiring diagram (WormWiring.org), we propose that cGMP then flows via gap junction connections from the site of its production in the ODR-1-expressing AWB/AWC/ASI sensory neurons, through ADF, AFD and AIA, to the ASH nociceptors. Once in ASH, cGMP activates the cGMP-dependent protein kinase EGL-4, which likely directly phosphorylates the regulator of G protein signaling proteins RGS-2 and RGS-3, stimulating their activity [58]. RGS-2 and RGS-3 downregulate Gα proteins that signal downstream of G protein-coupled receptors (GPCRs) that are activated by ligands such as quinine and octanol. When animals are well-fed, cGMP levels are low and there is only minimal inhibition of G protein-coupled signaling in ASH. Upon food remove, cGMP influx into ASH activates EGL-4, resulting in diminished nociceptive behavioral sensitivity to a subset of ASH-detected aversive stimuli. Decentralized modulation of ASH sensitivity may allow an animal to integrate multiple environmental cues with its internal state to maximize the appropriateness of its response to its surroundings. (B) The same diagram depicted in Fig 5A is shown here, but with only those gap junction connections originally reported in White et al. [2] included. (C) The left column shows a list of the neurons reported by White et al. [2] to make gap junctions with ASH, while the right column lists the neurons currently annotated at WormWiring.org to make gap junctions with ASH. The neurons shown in bold are those that are common to both lists.

We speculate that food somehow suppresses ODR-1 activity in the upstream AWB, AWC and/or ASI sensory neurons, while removal of food allows for ODR-1 activation and cGMP accumulation (Fig 8A). ODR-1 is most similar to transmembrane guanylyl cyclases, which are often regulated by extracellular peptides [102]. However, we found that the extracellular domain is not required for ODR-1 function in modulating ASH sensitivity (Fig 1). In addition, while soluble (cytoplasmic) guanylyl cyclases are generally activated by nitric oxide [103], *C*. *elegans* lack a nitric oxide synthase. Thus, the mechanistic link between an animal’s feeding status and ODR-1 activity is unclear. Interestingly, calcium levels rise in the AWCs upon withdrawal of either odorant or bacterial-conditioned media [104]. An attractive possibility is that this increase in calcium could directly or indirectly activate ODR-1 activity in the AWCs, which would provide a link between food withdrawal, cGMP generation in this neuron pair and the downstream dampening of ASH-mediated responses. Alternatively, ODR-1 may be constitutively active and phosphodiesterase activity may be regulated by an animal’s feeding status to adjust the pool of available cGMP.

Collectively, our data support a model wherein, upon food removal, the ODR-1-expressing neurons AWB, AWC and ASI provide a pool of cGMP that flows through a gap junction network from the site of its production in these sensory neurons, through ADF, AFD and AIA, to the ASH nociceptors (Fig 8A). However, the gap junctions reported in the wiring diagram first published by White et al. [2] do not provide a straightforward explanation for our experimental observations (Fig 8B). For example, White et al. [2] did not report gap junctions between ASH and ADF, AFD or AIA (Fig 8C). Therefore, these neurons were not predicted to connect the ODR-1-expressing neurons (AWB, AWC and ASI) to ASH via a gap junction circuit (Fig 8B) [2]. For our study, we referred to the re-annotated wiring diagram, which is based on the original electron micrograph series [2] and curated at WormWiring.org (Scott Emmons, personal communication) (Fig 5A). If the presence of the newly annotated gap junctions is not corroborated by future analysis, then alternate pathways/circuits for the non-cell-autonomous role of ODR-1 in the regulation of ASH-mediated nociceptive sensory behaviors must be considered.

INX-4 function in the ASHs is sufficient to rescue the quinine hypersensitivity of *inx-4(lof)* animals (Fig 3), suggesting that INX-4 expressed in ASH likely forms heterotypic gap junctions with an unidentified innexin(s) in ADF/AFD/AIA. We note that the reported expression patterns for most of the innexins were determined using only short upstream regulatory sequences to drive GFP expression [35]. Therefore, it is likely that the full expression pattern of each innexin is not yet known. For example, while the 706 bp upstream promoter for *inx-19* (also known as *nsy-5*) drove GFP expression in the AVBs and AVKs [35], a 5.8 kb *inx-19* promoter did not express in these neurons but instead was seen to drive GFP expression in 18 other neuron pairs (including AWB, AWC, ASI, ADF, AFD and ASH) [105]. Thus, further analysis is needed to correlate endogenous innexin expression with the anatomically defined gap junctions present throughout the *C*. *elegans* nervous system. While our combination of cell-specific rescue and ablation experiments strongly supports a role for the proposed gap junction network in the modulation of ASH activity, it is possible that extracellular cGMP could also enter ASH through an INX-4 hemichannel.

Delayed delivery of cGMP to the ASHs, due to flux through such a network, may help to optimize an animal’s navigation through an unpredictable environment [106]. Additionally, since *C*. *elegans* lack a complex central nervous system with which to simultaneously process several sets of sensory information, a gap junction network may allow for the efficient integration of diverse signals representing the environmental landscape with an animal’s experiences and internal state. This may allow for a more sophisticated modulation of behavior than could be achieved by expressing a guanylyl cyclase(s) directly in the ASHs themselves, especially given that the ASHs detect multiple forms of aversive stimuli [46–54]. Consistent with this possibility, we found that *odr-1(lof)* animals are hypersensitive in their response to some (e.g. quinine), but not all (primaquine, SDS, copper), ASH-detected stimuli (Fig 1, S3 Fig).

ODR-1 is not the only guanylyl cyclase that appears to downregulate ASH-mediated behavioral responses. We previously reported that animals lacking GCY-27, GCY-33 or GCY-34 function are also hypersensitive in their avoidance of dilute quinine [58]. GCY-27 is expressed in the ASJ, ASK and ASI head sensory neurons, although its role in these cells has not been characterized [59]. GCY-33 and GCY-34 contribute to the detection of oxygen levels by the BAG neurons and the URX, AQR, and PQR neurons, respectively [107–112]. Oxygen detection has been hypothesized to be important for *C*. *elegans* because aerobic bacteria, which utilize oxygen for metabolic processes, serve as a food source for animals in their native soil environment. Therefore, *C*. *elegans* may correlate low ambient oxygen levels with locations having nutritive potential. Thus, guanylyl cyclase function in neurons besides AWB/AWC/ASI could also serve to modulate ASH sensitivity based on food availability cues. Future studies focused on GCY-27, GCY-33 and GCY-34 are needed to shed light on the mechanism by which these guanylyl cyclases ultimately influence ASH-mediated behavioral responses. If they regulate ASH function non-cell-autonomously, in a manner similar to ODR-1, this would allow for the integration of an even greater array of sensory information to generate animal behavior that is optimized based on environment and experience.

Gap junctions have been classically described for their role in electrical coupling of neurons [113,114]. However, it is now appreciated that a variety of ions and small molecules can pass through these channels [24–32]. In mammalian systems, changes in gap junction protein expression have been documented in central nervous system pathologies [115,116], and studies in cultured cells have suggested possible opposing contributions for gap junctions. For example, while they may play a neuroprotective role in brain ischemia, gap junctions appear to contribute to the propagation of excitatory activity in epilepsy [115–117]. With a compact nervous system and a well-characterized behavioral repertoire, *C*. *elegans* serves as an excellent animal model to study the *in vivo* roles of gap junctions and their contribution to circuit-level modulation of neuronal function.

## Materials and Methods

### *C*. *elegans* Culture

Strains were maintained under standard conditions on NGM agar plates seeded with OP50 *E*. *coli* bacteria [118]. For a list of strains used in this study, see Supplemental Information.

### Behavioral Assays

Well-fed young adults were used for analysis, and all behavioral assays were performed on at least three separate days, in parallel with controls. Response to the soluble aversive tastants was scored as the percentage of animals that initiated backward locomotion within 4 seconds of encountering a drop of the tastant placed on the agar plate in front of a forward moving animal [51,52,119]. Tastants were dissolved in M13, pH 7.4 [120]. For soluble tastant avoidance assays, animals were tested 30 minutes after transfer to NGM plates lacking bacteria (“off food”). Response to octanol was scored as the amount of time it took an animal to initiate backward locomotion when presented with a hair dipped in octanol [48,80]. The nonanone avoidance assays were performed essentially as described [66] with the following modifications: round 100x15 mm plates were used with 14 ml of agar and animals were incubated on the nonanone plates for two hours. The Avoidance Index (A.I.) was calculated as the number of animals in sectors A and B, minus the number of animals in sectors E and F, divided by the total number of animals (not including animals that did not move from their original placement) [66]. The benzaldehyde chemotaxis assays were performed as previously described [60]. After one hour, the Chemotaxis Index (C.I.) was calculated as the number of animals that had accumulated at the attractant, minus the number of animals at the control, divided by the total number of animals [60]. For heat shock experiments, animals were raised to young adulthood and then shifted to 33°C for two hours. They were allowed to recover for four hours at 20°C prior to assaying. All data is presented as ± standard error of the mean (SEM). The Student’s two-tailed t-Test and one-way Anova with Tukey’s Honestly Significant Difference (HSD) were used for statistical analyses. In all figures, * denotes p < 0.05, ** denotes p < 0.001, and *** denotes p < 0.0001. n.s. denotes p ≥ 0.05.

## Supporting Information

S1 Fig Confirmation of loss of synaptic transmission in AWB and AWC. (A) The loss of UNC-13-dependent synaptic signaling from the AWB head sensory neurons resulted in failure to avoid the aversive odorant 2-nonanone, which is detected by the AWBs [66]. The *str-1* (AWB) [66] promoter was used to co-express a non-coding fragment of *unc-13* in both the sense and antisense orientations of otherwise wild-type animals. RNAi knock-down of *unc-13* in the AWB neurons resulted in diminished avoidance of a 1:10 dilution of 2-nonanone (p < 0.0001 when compared to wild-type animals). (B) Loss of UNC-13-dependent synaptic signaling from the AWC head sensory neurons resulted in failure to chemotax towards benzadehyde, which is detected by the AWCs [60]. The *ceh-36p3* (AWC) [65], *gpa-4* (ASI) [64] and *str-1p* (AWB) [66] promoters were used to co-express a non-coding fragment of *unc-13* in both the sense and antisense orientations of otherwise wild-type animals. RNAi knock-down of *unc-13* in the AWC neurons or in the AWB, AWC or ASI neurons in combination abolished chemotaxis to a 1:200 dilution of benzaldehyde. The combined data of ≥ 3 independent lines and n ≥ 120 transgenic animals is shown in each panel. Avoidance index = ((A + B)–(E + F)) ÷ total number of animals on the assay plate [66]. Chemotaxis index = (number of animals at odorant − number of animals at control) ÷ total number of animals on the assay plate [60]. Alleles used: *odr-1(n1936)* loss-of-function and *inx-4(ok2373)* loss-of-function. WT = the N2 wild-type strain. lof = loss-of-function.
(TIF)

S2 Fig INX-4 is expressed in larvae and adult animals. The *inx-4* promoter [35] was used to drive expression of GFP (*inx-4p*::*gfp*). Transgenic animals were incubated with the lipophilic dye DiD (shown in red), which is taken up by the ASH sensory neurons and was used to visualize the neuronal cell body. As previously reported [35], *inx-4* expression was seen in the ASH sensory neurons of larval-stage animals, including the L2 stage (top panel). In addition, *inx-4* expression was observed in the ASHs of adult animals (bottom panel).
(TIF)

S3 Fig ODR-1 and INX-4 do not regulate ASH sensitivity in general. *C*. *elegans* respond to aversive stimuli in addition to quinine. (A-C) *odr-1(lof)* and *inx-4(lof)* animals responded similarly to wild-type animals to the bitter tastant primaquine, the detergent SDS and the heavy metal copper, across a range of concentrations (p > 0.2 for each concentration). The percentage of animals responding is shown. n ≥ 60 for each. All tastants were dissolved in M13 buffer, pH 7.4. Alleles used: *inx-4(ok2373)* loss-of-function and *odr-1(n1936)* loss-of-function. WT = the N2 wild-type strain. lof = loss-of-function.
(TIF)

S4 Fig cGMP generation in ASH dampens quinine sensitivity. The ASH-selective *osm-10* [48] and *srb-6* [80] promoters were used to drive expression of a blue light-inducible guanylyl cyclase (BlgC) or blue light-inducible adenylyl cyclase (BlaC) [81] in *lite-1(lof)* animals. *osm-10p* drives expression in ASH and weakly in ASI in the head, and in PHA and PHB in the tail. *srb-6p* drives expression in the ASH, ADL and ADF head sensory neurons, and in PHA and PHB in the tail. Adult *lite-1(lof)* animals expressing BlgC or BlaC were tested without blue light exposure (white bars) or after a 30 second exposure (grey bars). While *lite-1(lof)* animals responded robustly to 5 mM quinine, similar to WT animals (p > 0.2), transgenic animals expressing BlgC displayed a significantly diminished response following blue light exposure (p < 0.0001 for each ASH-selective promoter). Transgenic animals expressing BlaC remained sensitive following blue light exposure (p > 0.1 when compared to wild-type animals). The percentage of animals responding is shown. The combined data of ≥ 3 independent lines and n ≥ 120 transgenic animals is shown in each panel. Allele used: *lite-1(ce314)* loss-of-function. WT = the N2 wild-type strain. lof = loss-of-function.
(TIF)

S5 Fig cGMP generation in ADF dampens quinine sensitivity. The ADF-specific *srh-142* promoter [79] was used to drive expression of a blue light-inducible guanylyl cyclase (BlgC) or blue light-inducible adenylyl cyclase (BlaC) [81] in *lite-1(lof)* or *inx-4(lof);lite-1(lof)* animals. Adult *lite-1(lof)* or *inx-4(lof);lite-1(lof)* animals expressing BlgC or BlaC were tested without blue light exposure (white bars) or after a 30 second exposure and 30 second recovery (grey bars). While *lite-1(lof)* animals responded robustly to 10 mM quinine, similar to WT animals (p > 0.1), transgenic animals expressing BlgC in the ADFs displayed a significantly diminished response following blue light exposure (p < 0.001). Transgenic animals expressing BlaC did not display a diminished response following blue light exposure (p > 0.1 when compared to wild-type animals). *inx-4(lof);lite-1(lof)* transgenic animals expressing either BlgC or BlaC did not display a diminished response following blue light exposure (p > 0.5 when compared to wild-type animals). The percentage of animals responding is shown. The combined data of ≥ 3 independent lines and n ≥ 120 transgenic animals is shown in each panel. Alleles used: *lite-1(ce314)* loss-of-function and *inx-4(ok2373)* loss-of-function. WT = the N2 wild-type strain. lof = loss-of-function.
(TIF)

S1 Text Supporting text. (DOCX)

S1 Table Transgenic strains used in the study. (DOCX)
