# Supplementary figures and images for: Aversive Behavior in the Nematode C. elegans Is Modulated by cGMP and a Neuronal Gap Junction Network
Source: PLoS Genet. 2016 Jul 26;12(7):e1006153. doi: 10.1371/journal.pgen.1006153 (PMC4961389; doi:10.1371/journal.pgen.1006153)

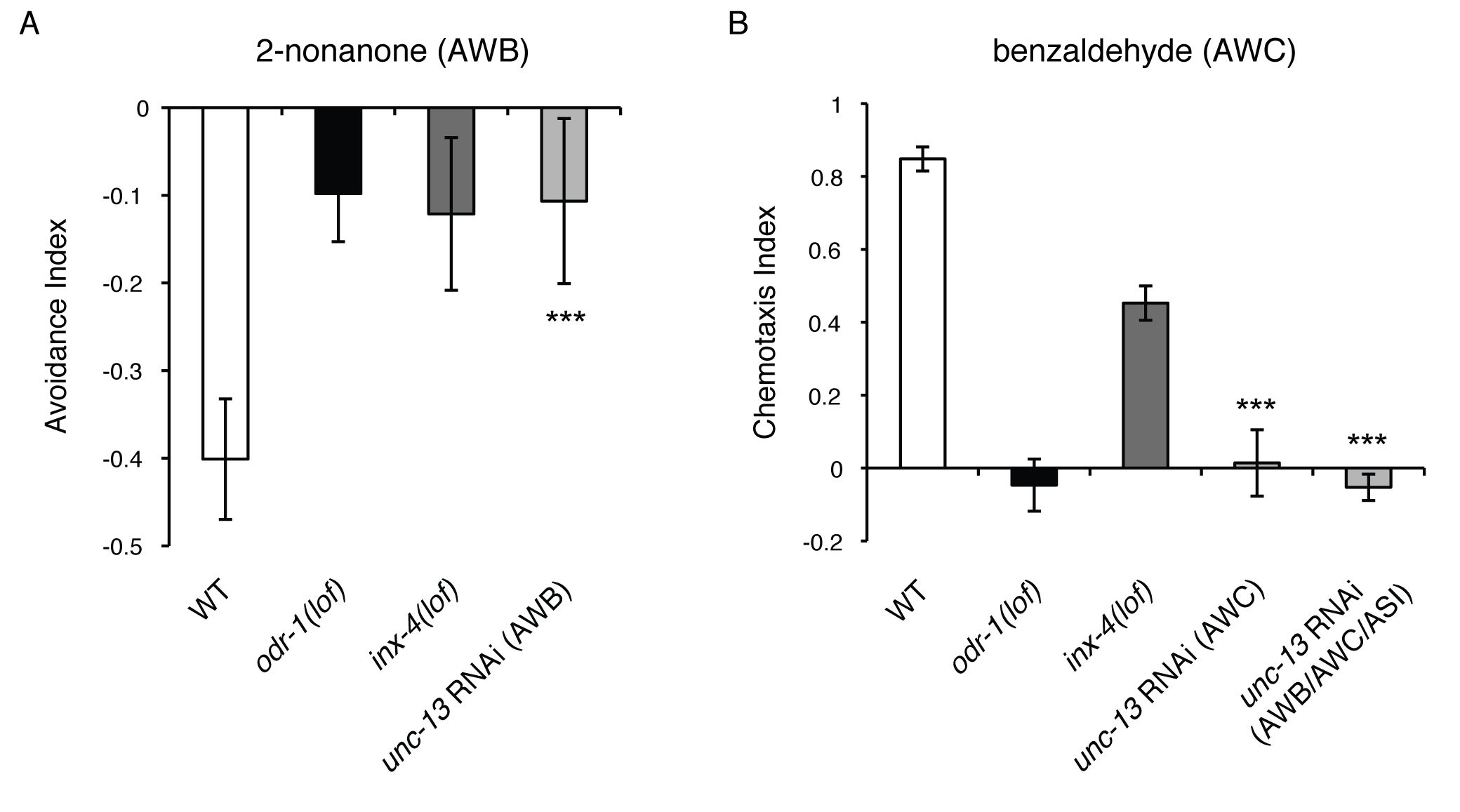

Supplement: S1 Fig — (A) The loss of UNC-13-dependent synaptic signaling from the AWB head sensory neurons resulted in failure to avoid the aversive odorant 2-nonanone, which is detected by the AWBs [66]. The str-1 (AWB) [66] promoter was used to co-express a non-coding fragment of unc-13 in both the sense and antisense orientations of otherwise wild-type animals. RNAi knock-down of unc-13 in the AWB neurons resulted in diminished avoidance of a 1:10 dilution of 2-nonanone (p < 0.0001 when compared to wild-type animals). (B) Loss of UNC-13-dependent synaptic signaling from the AWC head sensory neurons resulted in failure to chemotax towards benzadehyde, which is detected by the AWCs [60]. The ceh-36p3 (AWC) [65], gpa-4 (ASI) [64] and str-1p (AWB) [66] promoters were used to co-express a non-coding fragment of unc-13 in both the sense and antisense orientations of otherwise wild-type animals. RNAi knock-down of unc-13 in the AWC neurons or in the AWB, AWC or ASI neurons in combination abolished chemotaxis to a 1:200 dilution of benzaldehyde. The combined data of ≥ 3 independent lines and n ≥ 120 transgenic animals is shown in each panel. Avoidance index = ((A + B)–(E + F)) ÷ total number of animals on the assay plate [66]. Chemotaxis index = (number of animals at odorant − number of animals at control) ÷ total number of animals on the assay plate [60]. Alleles used: odr-1(n1936) loss-of-function and inx-4(ok2373) loss-of-function. WT = the N2 wild-type strain. lof = loss-of-function. (TIF) [file pgen.1006153.s001.tif]

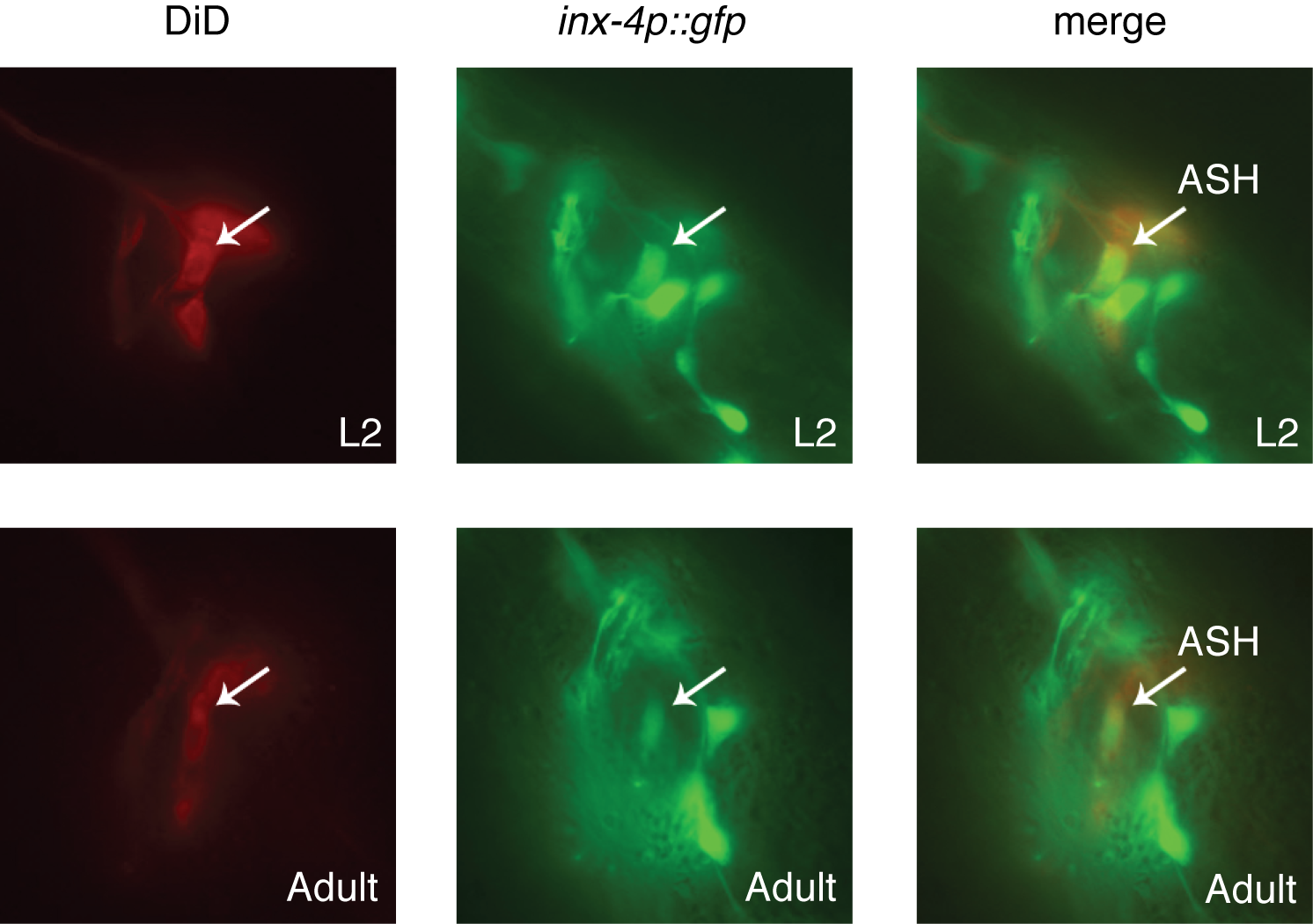

Supplement: S2 Fig — The inx-4 promoter [35] was used to drive expression of GFP (inx-4p::gfp). Transgenic animals were incubated with the lipophilic dye DiD (shown in red), which is taken up by the ASH sensory neurons and was used to visualize the neuronal cell body. As previously reported [35], inx-4 expression was seen in the ASH sensory neurons of larval-stage animals, including the L2 stage (top panel). In addition, inx-4 expression was observed in the ASHs of adult animals (bottom panel). (TIF) [file pgen.1006153.s002.tif]

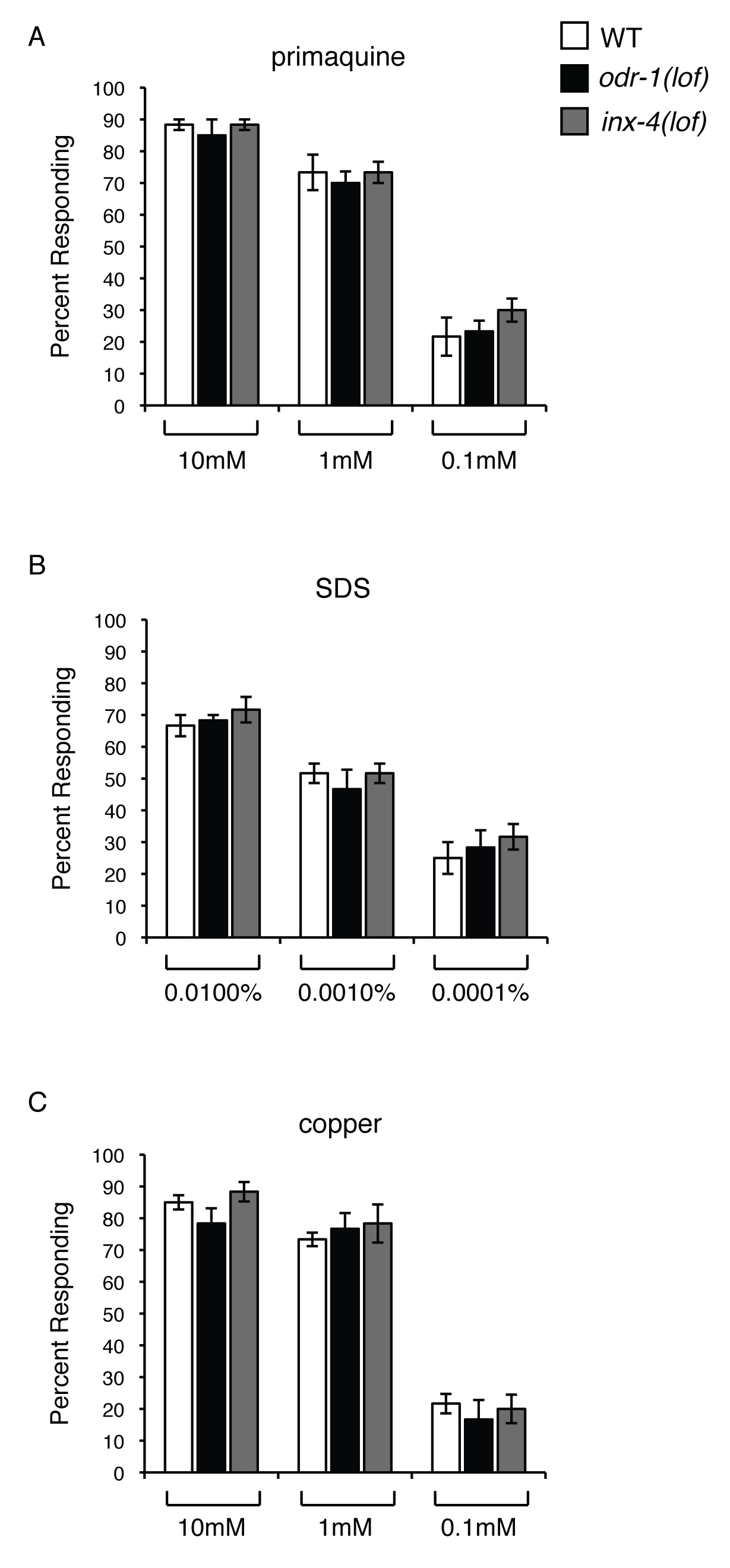

Supplement: S3 Fig — C. elegans respond to aversive stimuli in addition to quinine. (A-C) odr-1(lof) and inx-4(lof) animals responded similarly to wild-type animals to the bitter tastant primaquine, the detergent SDS and the heavy metal copper, across a range of concentrations (p > 0.2 for each concentration). The percentage of animals responding is shown. n ≥ 60 for each. All tastants were dissolved in M13 buffer, pH 7.4. Alleles used: inx-4(ok2373) loss-of-function and odr-1(n1936) loss-of-function. WT = the N2 wild-type strain. lof = loss-of-function. (TIF) [file pgen.1006153.s003.tif]

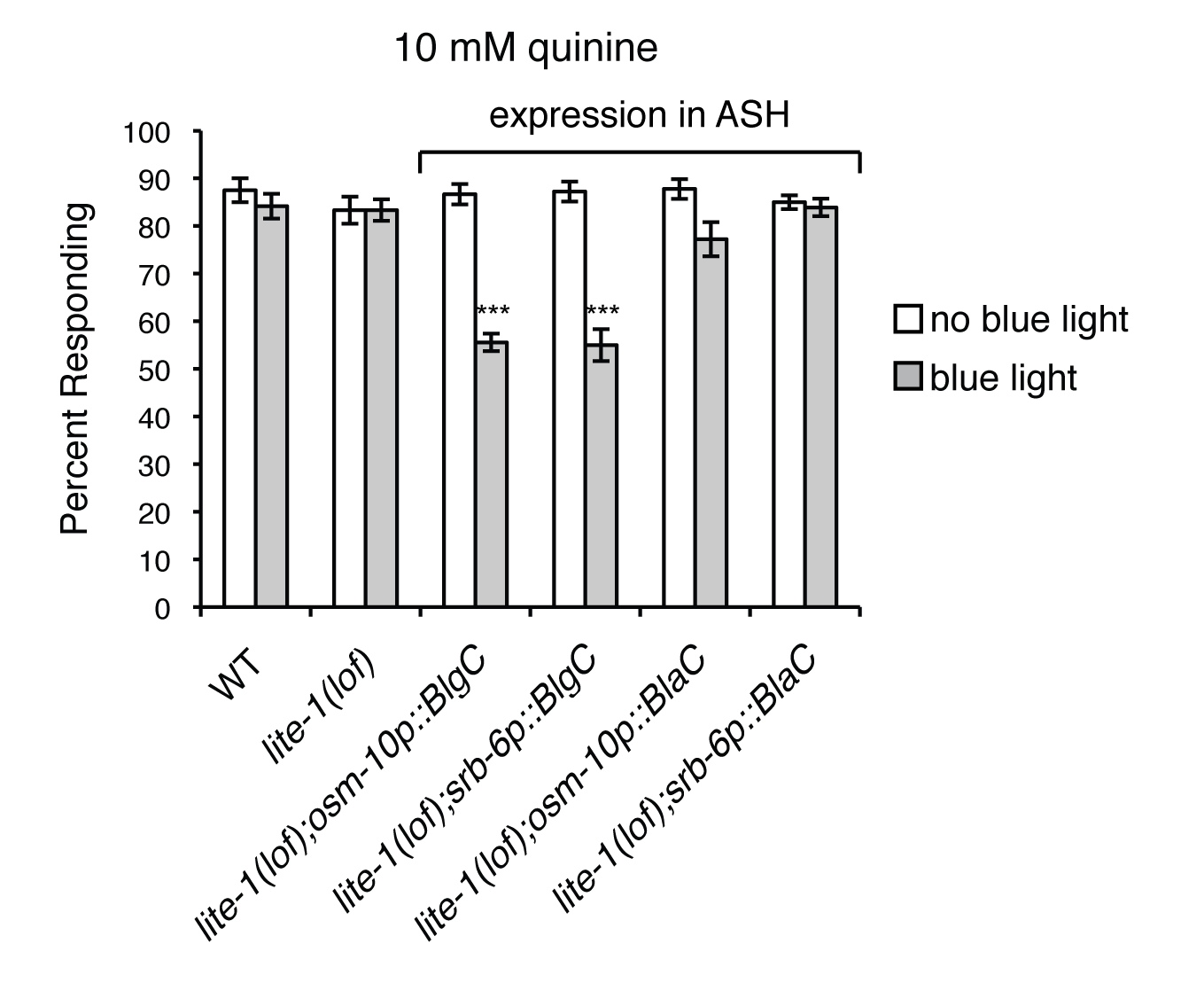

Supplement: S4 Fig — The ASH-selective osm-10 [48] and srb-6 [80] promoters were used to drive expression of a blue light-inducible guanylyl cyclase (BlgC) or blue light-inducible adenylyl cyclase (BlaC) [81] in lite-1(lof) animals. osm-10p drives expression in ASH and weakly in ASI in the head, and in PHA and PHB in the tail. srb-6p drives expression in the ASH, ADL and ADF head sensory neurons, and in PHA and PHB in the tail. Adult lite-1(lof) animals expressing BlgC or BlaC were tested without blue light exposure (white bars) or after a 30 second exposure (grey bars). While lite-1(lof) animals responded robustly to 5 mM quinine, similar to WT animals (p > 0.2), transgenic animals expressing BlgC displayed a significantly diminished response following blue light exposure (p < 0.0001 for each ASH-selective promoter). Transgenic animals expressing BlaC remained sensitive following blue light exposure (p > 0.1 when compared to wild-type animals). The percentage of animals responding is shown. The combined data of ≥ 3 independent lines and n ≥ 120 transgenic animals is shown in each panel. Allele used: lite-1(ce314) loss-of-function. WT = the N2 wild-type strain. lof = loss-of-function. (TIF) [file pgen.1006153.s004.tif]

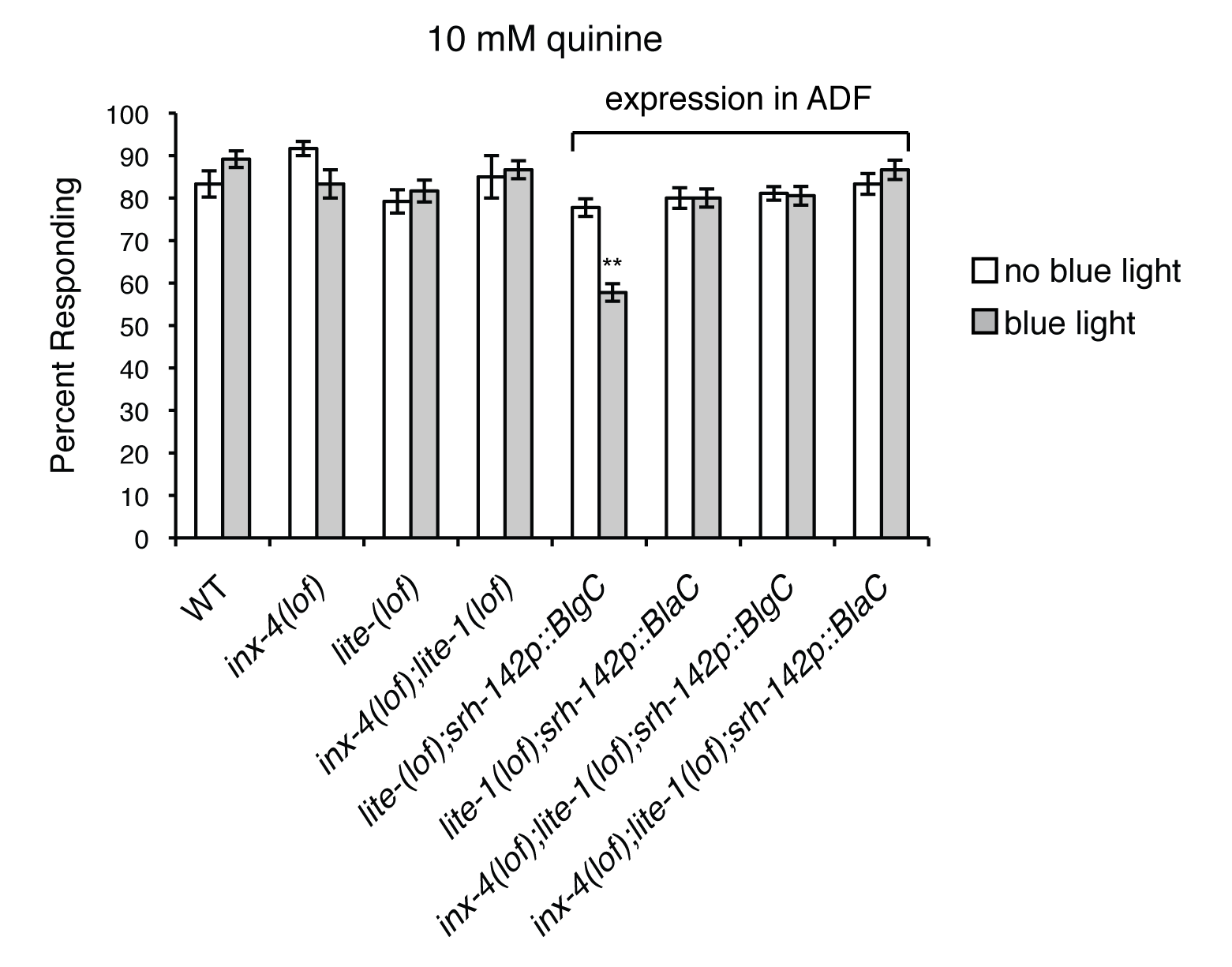

Supplement: S5 Fig — The ADF-specific srh-142 promoter [79] was used to drive expression of a blue light-inducible guanylyl cyclase (BlgC) or blue light-inducible adenylyl cyclase (BlaC) [81] in lite-1(lof) or inx-4(lof);lite-1(lof) animals. Adult lite-1(lof) or inx-4(lof);lite-1(lof) animals expressing BlgC or BlaC were tested without blue light exposure (white bars) or after a 30 second exposure and 30 second recovery (grey bars). While lite-1(lof) animals responded robustly to 10 mM quinine, similar to WT animals (p > 0.1), transgenic animals expressing BlgC in the ADFs displayed a significantly diminished response following blue light exposure (p < 0.001). Transgenic animals expressing BlaC did not display a diminished response following blue light exposure (p > 0.1 when compared to wild-type animals). inx-4(lof);lite-1(lof) transgenic animals expressing either BlgC or BlaC did not display a diminished response following blue light exposure (p > 0.5 when compared to wild-type animals). The percentage of animals responding is shown. The combined data of ≥ 3 independent lines and n ≥ 120 transgenic animals is shown in each panel. Alleles used: lite-1(ce314) loss-of-function and inx-4(ok2373) loss-of-function. WT = the N2 wild-type strain. lof = loss-of-function. (TIF) [file pgen.1006153.s005.tif]
