# Supplementary material for: Aversive Behavior in the Nematode C. elegans Is Modulated by cGMP and a Neuronal Gap Junction Network
Source: PLoS Genet. 2016 Jul 26;12(7):e1006153. doi: 10.1371/journal.pgen.1006153 (PMC4961389; doi:10.1371/journal.pgen.1006153)
Supplement: S1 Text — (DOCX) [file pgen.1006153.s006.docx]

**S1 Text:**

**Strains**

Strains used in this study include: N2 Bristol wild-type, MT1074 *egl-4(n479)*, FG290 *odr-1(n1936)*, FG614 *inx-4(ok2373)*, FG615 *inx-4(ok2373);odr-1(n1936)*, FG613 *inx-20(ok426)*, LX242 *rgs-3(vs19)*, KG1180 *lite-1(ce314)*, FG725 *inx-4(ok2373);lite-1(ce314)*, FG726 *inx-20(426);inx-4(ok2373)*, PY7502 *oyIs85* *(ceh-36p::TU#813 + ceh-36p::TU#814 + srtx-1p::GFP + unc-122p::dsRed)*, PY7505 *oyIs84 (gpa-4p::TU#813 + gcy-27p::TU#814 + gcy-27p::GFP + unc-122p::dsRed)*, PY7507 *oyIs85;oyIs84*, GN112 *pgIs2 (gcy-8p::TU#813 + gcy-8p::TU#814 + unc-122p::GFP + gcy-8p::mCherry + gcy-8p::GFP + ttx-3p::GFP)*. Additional strains used for the innexin candidate gene screen include: DA1402 *eat-5(ad1402)*, VC260 *inx-2(ok376)*, RB1834 *inx-4(ok2373)*, RB1086 *inx-5(ok1053)*, MR127 *inx-6(rr5)*, RB1792 *inx-7(ok2319)*, VC116 *inx-8(gk42)*, FG612 *inx-8(gk42)*, VC994 *inx-9(ok1502)*, RB2051 *inx-10(ok2714)*, RB2108 *inx-11(ok2783)*, AU98 *inx-14(ag17)*, RB1896 *inx-18(ok2454)*, CX6161 *inx-19(ky634)*, RB683 *inx-20(ok426)*, RB1929 *inx-21(ok2524)*, XM1011 *inx-22(tm1661)*. The *inx-16* mutant was not available at the time of the screen. Loss-of-function animals for three innexins (*unc-7a*, *unc-7b* and *unc-9*) are uncoordinated, making movement-based behavioral assays infeasible. Viable homozygous loss-of-function alleles are not available for the remaining innexins (*inx-1*, *inx-3*, *inx-12*, *inx-13*, *inx-15* and *inx-17*).

**Transgenic Strains**

Germline transformations were performed as previously described [1]. For *odr-1* and *inx-4* rescue experiments, 25 ng/μl of pJM67 *elt-2::gfp* plasmid [2] or 15 ng/μl of pELA1 *vha-6p::mCherry* [3] was used as the co-injection marker, along with 50 ng/μl of the rescuing plasmid. Cell-specific RNAi knock-down experiments were performed as previously described [4]. 25 ng/μl of pJM67 *elt-2::gfp* plasmid [2] or 15 ng/μl of pELA1 *vha-6p::mCherry* [3] was co-injected with 50 ng/μl of each PCR fusion product [4]. For the light-inducible cyclase experiments, 15 ng/μl of pELA1 *vha-6p::mCherry* [3] was used as the co-injection marker, along with 20 ng/μl of the cyclase plasmid and 65 ng/μl of pUC19 [5]. Genetic ablation experiments were performed as previously described **[6].** 25 ng/μl of pJM67 *elt-2::gfp* plasmid [2] or 15 ng/μl of pELA1 *vha-6p::mCherry* [3] was co-injected with 50 ng/μl of recCaspase expressing constructs and 25ng/μl of cell-specific GFP or mCherry constructs to mark neurons. In all experiments, we selected for the most stable arrays (based on the co-injection marker) and assayed > 5 transgenic lines resulting from each injection. The data from the three lines with the strongest phenotype were pooled for analysis in each case. See also S1 Table.

**Plasmid Construction**

pFG1: The ~900 bp *osm-10* promoter was isolated from CR142 [7] using PstI/BamHI and inserted into these sites of Fire vector pPD49.26 (Fire Lab *C. elegans* Vector Kit, Addgene).

pFG50: The ~2.1 kb *srbc-66* promoter was isolated from *srbc-66::gfp* [8] using HindIII/BamHI and inserted into these sites of Fire vector pPD49.26 (Fire Lab *C. elegans* Vector Kit, Addgene).

pFG140 *osm-10p::odr-1*: The ~6 kb *odr-1* genomic fragment was PCR amplified from KC3 [9], incorporating a 5’ NheI site and a 3’ NcoI site, and subcloned into these sites of pFG1.

pFG141 *srb-6p::odr-1*: The ~6 kb *odr-1* genomic fragment was isolated from pFG140 using NheI/NcoI and inserted into these sites of pFG10 [10].

pFG148: The ~2.2 kb *gcy-8* promoter was isolated from *gcy-8 in MC10* (gift from Piali Sengupta) using SphI/BamHI and inserted into these sites of Fire vector pPD49.26 (Fire Lab *C. elegans* Vector Kit, Addgene).

pFG149: The ~3 kb *gcy-28d* promoter was isolated from *gcy-28d::GCaMP5* (gift from Cori Bargmann) using SphI/BamHI and inserted into these sites of Fire vector pPD49.26 (Fire Lab *C. elegans* Vector Kit, Addgene).

pFG150: The ~3 kb *srh-142* promoter was isolated from *srh-142::dsRed* [11] using SphI/BamHI and inserted into these sites of Fire vector pPD49.26 (Fire Lab *C. elegans* Vector Kit, Addgene).

pFG193: The ~3 kb *str-1* promoter [12,13] was isolated from *str-1 in pSM6.1* (gift from Piali Sengupta) using PstI/BamHI and inserted into these sites of Fire vector pPD49.26 (Fire Lab *C. elegans* Vector Kit, Addgene).

pFG207: The ~2.8 kb *gpa-4* promoter [14] was isolated from *gpa-4p in pMC10* (gift from Piali Sengupta) using HindIII/BamHI and inserted into these sites of Fire vector pPD49.26 (Fire Lab *C. elegans* Vector Kit, Addgene).

pFG205: The ~350 bp *ceh-36p3* promoter [15] was isolated from *ceh-36p3::mCherry* (L’Etoile Lab) using HindIII/BamHI and inserted into these sites of Fire vector pPD49.26 (Fire Lab *C. elegans* Vector Kit, Addgene).

pFG206 *str-1p::odr-1*: Genomic *odr-1* sequence was isolated from pFG140 using BamHI/ApaI and inserted into these sites of pFG193.

pFG204 *ceh-36p3::odr-1*: Genomic *odr-1* sequence was isolated from pFG140 using BamHI/ApaI and inserted into these sites of pFG205.

pFG208 *gpa-4p::odr-1*: Genomic *odr-1* sequence was isolated from pFG141 using BamHI/ApaI and subcloned into these sites of pFG207.

pFG170 *srbc-66p::odr-1*: Genomic *odr-1* sequence was isolated from pFG141 using NheI/ApaI and subcloned into these sites of pFG50.

pFG209 *hsp::odr-1*: Genomic *odr-1* sequence was isolated from pFG141 using BamHI/ApaI and inserted into these sites of Fire vector pPD49.78 (Fire Lab *C. elegans* Vector Kit, Addgene).

pFG172 *osm-10p::ced-3(p15)::nz*: The ~900bp *osm-10* promoter was isolated from pFG1 using HindII/BamHI and inserted into these sites of [*Pmec-18 ced-3 (p15)::nz* [TU#806]](https://www.addgene.org/16080/) (Chalfie Lab, Addgene vector #16080 [6]), replacing the *mec-18* promoter.

pFG175 *srb-6p::ced-3(p17)::cz*: *ced-3(p17)::cz* sequence was isolated from *Pmec-18 cz::ced-3 (p17)* [TU#807] (Chalfie Lab, Addgene vector #16081 [6]) using BamHI/ApaI and subcloned into these sites of pFG10 [10].

pFG187 *srbc-66p::ced-3(p15)::nz*: The ~2.1 kb *srbc-66* promoter was isolated from pFG50 using HindII/BamHI and inserted into these sites of [*Pmec-18 ced-3 (p15)::nz* [TU#806]](https://www.addgene.org/16080/) (Chalfie Lab, Addgene vector #16080 [6]), replacing the *mec-18* promoter.

pFG188 *srbc-66p::ced-3(p17)::cz*: The ~2.1 kb *srbc-66* promoter was isolated from pFG50 using HindII/BamHI and inserted into these sites of *Pmec-18 cz::ced-3 (p17)* [TU#807] (Chalfie Lab, Addgene vector #16081 [6]), replacing the *mec-18* promoter.

pFG186 *srbc-66p::gfp*: The ~2.1 kb *srbc-66* promoter was isolated from pFG50 using HindIII/BamHI and inserted into these sites of pPD95.75 (Fire Lab *C. elegans* Vector Kit, Addgene).

pFG173 *gcy-8p::ced-3(p15)::nz*: *ced-3(p15)::nz* sequence was isolated from [*Pmec-18 ced-3 (p15)::nz* [TU#806]](https://www.addgene.org/16080/) (Chalfie Lab, Addgene vector #16080 [6]) using BamHI/ApaI and subcloned into these sites of pFG148.

pFG180 *gcy-28dp::ced-3(p15)::nz*: *ced-3(p15)::nz* sequence was isolated from [*Pmec-18 ced-3 (p15)::nz* [TU#806]](https://www.addgene.org/16080/) (Chalfie Lab, Addgene vector #16080 [6]) using BamHI/ApaI and inserted into these sites of pFG149.

pFG181 *gcy-28dp::ced-3(p17)::cz*: *ced-3(p17)::cz* sequence was isolated from *Pmec-18 cz::ced-3 (p17)* [TU#807] (Chalfie Lab, Addgene vector #16081 [6]) using BamHI/ApaI and inserted into these sites of pFG149.

pFG183 *gcy-28dp::gfp*: *gfp* sequence was isolated from pPD95.75 (Fire Lab *C. elegans* Vector Kit, Addgene) using BamHI/ApaI and inserted into these sites of pFG149.

pFG190 *srh-142p::ced-3(p15)::nz*: *ced-3(p15)::nz* sequence was isolated from [*Pmec-18 ced-3 (p15)::nz* [TU#806]](https://www.addgene.org/16080/) (Chalfie Lab, Addgene vector #16080 [6]) using BamHI/ApaI and inserted into these sites of pFG150.

pFG191 *srh-142p::ced-3(p17)::cz*: *ced-3(p17)::cz* sequence was isolated from *Pmec-18 cz::ced-3 (p17)* [TU#807] (Chalfie Lab, Addgene vector #16081 [6]) using BamHI/ApaI and inserted into these sites of pFG150.

pFG184 *srh-142p::gfp*: *gfp* sequence was isolated from pPD95.75 (Fire Lab *C. elegans* Vector Kit, Addgene) using BamHI/ApaI and inserted into these sites of pFG150.

pFG194 *str-1p::ced-3(p15)::nz*: *ced-3(p15)::nz* sequence was isolated from [*Pmec-18 ced-3 (p15)::nz* [TU#806]](https://www.addgene.org/16080/) (Chalfie Lab, Addgene vector #16080 [6]) using BamHI/ApaI and inserted into these sites of pFG193.

pFG195 *str-1p::ced-3(p17)::cz*: *ced-3(p17)::cz* sequence was isolated from *Pmec-18 cz::ced-3 (p17)* [TU#807] (Chalfie Lab, Addgene vector #16081 [6]) using BamHI/ApaI and inserted into these sites of pFG193.

pFG196 *str-1p::gfp*: The ~3 kb *str-1* promoter [12,13] was isolated from *str-1* in pSM6.1 (gift from Piali Sengupta) using PstI/BamHI and inserted into these sites of pPD95.75 (Fire Lab *C. elegans* Vector Kit, Addgene).

pFG230 *aex-3::ced-3(p17)*: The ~1.3 kb *aex-3* promoter was isolated from pFG231 using HindIII/BamHI, and inserted into these sites of *Pmec-18 cz::ced-3 (p17)* [TU#807] (Chalfie Lab, Addgene vector #16081 [6]), replacing the *mec-18* promoter.

pFG231 *aex-3p::gfp*: The ~1.3 kb *aex-3* promoter was PCR amplified from N2 genomic DNA, incorporating a 5’ HindIII and a 3’ BamHI site, and subcloned into these sites of pPD95.75 (Fire Lab *C. elegans* Vector Kit, Addgene).

pFG211: The ~1.6 kb *inx-4* cDNA was synthesized by BioBasic and inserted into the NheI/Asp718 sites of pPD49.26 (Fire Lab *C. elegans* Vector Kit, Addgene).

pFG212 *osm-10p::inx-4*: The ~1.6 kb *inx-4* cDNA was isolated from pFG211 using NheI/Asp718 and inserted into these sites of pFG1.

pFG233: The ~1.2 kb *srd-10* promoter was PCR amplified from N2 genomic lysate, incorporating a 5’ PstI and 3’ BamHI site, and subcloned into these sites of pPD49.26 (Fire Lab *C. elegans* Vector Kit, Addgene).

pFG225 *srd-10p::inx-4*: The ~1.6 kb *inx-4* cDNA was isolated from pFG211 using NheI/Asp718 and inserted into these sites of pFG233.

pFG213: *srh-142p::inx-4*: The ~1.6 kb *inx-4* cDNA was isolated from pFG211 using NheI/ApaI and inserted into these sites of pFG150.

pFG210 *inx-4p::gfp*: The ~3.1 kb *inx-4* promoter was PCR amplified from N2 genomic lysate, incorporating a 5’ PstI and 3’ BamHI site, and subcloned into these sites of pPD95.75 (Fire Lab *C. elegans* Vector Kit, Addgene).

pFG226 *inx-4p::inx-4*: The ~3.1 kb *inx-4* promoter was isolated from pFG210 using PstI/BamHI and inserted into these sites of pFG211.

pFG232 *hsp::inx-4*: The ~1.6 kb *inx-4* cDNA was isolated from pFG211 using NheI/Asp718 and inserted into these sites of Fire vector pPD49.78 (Fire Lab *C. elegans* Vector Kit, Addgene).

pFG254 *odr-1p::inx-4*: The ~2.6 kb *odr-1* promoter was PCR amplified from KC3 [9], incorporating a 5’ BamHI site and a 3’ XmaI site, and subcloned into these sites of pFG211.

pFG221 *osm-10p::BlgC*: The ~1 kb *BlgC* DNA was PCR amplified from *MBP-His6-BlgC* in pMAL-c2x [16], incorporating a 5’ NheI site and a 3’ NcoI site, and subcloned into these sites of pFG1.

pFG222 *srb-6p::BlgC*: The ~1 kb *BlgC* DNA was isolated from pFG221 using NheI/NcoI and subcloned into these sites of pFG10 [10].

pFG223 *osm-10p::BlaC*: The ~1 kb *BlaC* DNA was PCR amplified from *MBP-His6-BlaC* in pMAL-c2x [16], incorporating a 5’ NheI site and a 3’ NcoI site, and subcloned into these sites of pFG1.

pFG224 *srb-6p::BlaC*: The ~1 kb *BlaC* DNA was isolated from pFG223 using NheI/NcoI and subcloned into these sites of pFG10 [10].

pFG242 *srh-142p::BlgC*: The ~1 kb *BlgC* DNA was isolated from pFG221 using NheI/ApaI and subcloned into these sites of pFG150.

pFG243 *srh-142p::BlaC*: The ~1 kb *BlaC* DNA was isolated from pFG223 using NheI/ApaI and subcloned into these sites of pFG150.

All constructs were verified by sequencing when appropriate.

**Supplemental References:**

1. Mello CC, Kramer JM, Stinchcomb D, Ambros V (1991) Efficient gene transfer in *C.elegans*: extrachromosomal maintenance and integration of transforming sequences. Embo J 10: 3959-3970.

2. Fukushige T, Hawkins MG, McGhee JD (1998) The GATA-factor *elt-2* is essential for formation of the *Caenorhabditis elegans* intestine. Dev Biol 198: 286-302.

3. Allman E, Johnson D, Nehrke K (2009) Loss of the apical V-ATPase a-subunit VHA-6 prevents acidification of the intestinal lumen during a rhythmic behavior in *C. elegans*. Am J Physiol Cell Physiol 297: C1071-1081.

4. Esposito G, Di Schiavi E, Bergamasco C, Bazzicalupo P (2007) Efficient and cell specific knock-down of gene function in targeted *C. elegans* neurons. Gene 395: 170-176.

5. Yanisch-Perron C, Vieira J, Messing J (1985) Improved M13 phage cloning vectors and host strains: nucleotide sequences of the M13mp18 and pUC19 vectors. Gene 33: 103-119.

6. Chelur DS, Chalfie M (2007) Targeted cell killing by reconstituted caspases. Proc Natl Acad Sci U S A 104: 2283-2288.

7. Rongo C, Whitfield CW, Rodal A, Kim SK, Kaplan JM (1998) LIN-10 is a shared component of the polarized protein localization pathways in neurons and epithelia. Cell 94: 751-759.

8. Kim K, Sato K, Shibuya M, Zeiger DM, Butcher RA, et al. (2009) Two chemoreceptors mediate developmental effects of dauer pheromone in *C. elegans*. Science 326: 994-998.

9. Krzyzanowski MC, Brueggemann C, Ezak MJ, Wood JF, Michaels KL, et al. (2013) The *C. elegans* cGMP-Dependent Protein Kinase EGL-4 Regulates Nociceptive Behavioral Sensitivity. PLoS Genet 9: e1003619.

10. Ezak MJ, Hong E, Chaparro-Garcia A, Ferkey DM (2010) *Caenorhabditis elegans* TRPV channels function in a modality-specific pathway to regulate response to aberrant sensory signaling. Genetics 185: 233-244.

11. Lanjuin A, VanHoven MK, Bargmann CI, Thompson JK, Sengupta P (2003) Otx/otd homeobox genes specify distinct sensory neuron identities in *C. elegans*. Dev Cell 5: 621-633.

12. Troemel ER, Kimmel BE, Bargmann CI (1997) Reprogramming chemotaxis responses: sensory neurons define olfactory preferences in *C. elegans*. Cell 91: 161-169.

13. Mukhopadhyay S, Lu Y, Qin H, Lanjuin A, Shaham S, et al. (2007) Distinct IFT mechanisms contribute to the generation of ciliary structural diversity in *C. elegans*. EMBO J 26: 2966-2980.

14. Jansen G, Thijssen KL, Werner P, van der Horst M, Hazendonk E, et al. (1999) The complete family of genes encoding G proteins of *Caenorhabditis elegans*. Nat Genet 21: 414-419.

15. Etchberger JF, Lorch A, Sleumer MC, Zapf R, Jones SJ, et al. (2007) The molecular signature and cis-regulatory architecture of a *C. elegans* gustatory neuron. Genes Dev 21: 1653-1674.

16. Ryu MH, Moskvin OV, Siltberg-Liberles J, Gomelsky M (2010) Natural and engineered photoactivated nucleotidyl cyclases for optogenetic applications. J Biol Chem 285: 41501-41508.
