# Supplementary material for: Aversive Behavior in the Nematode C. elegans Is Modulated by cGMP and a Neuronal Gap Junction Network
Source: PLoS Genet. 2016 Jul 26;12(7):e1006153. doi: 10.1371/journal.pgen.1006153 (PMC4961389; doi:10.1371/journal.pgen.1006153)
Supplement: S1 Table — (DOCX) [file pgen.1006153.s007.docx]

**S1 Table. List of Transgenic Strains**

| **FG580** *N2; udEx314[hsp::odr-1, elt-2p::gfp]* |
| --- |
| **FG581** *N2; udEx315[hsp::odr-1, elt-2p::gfp]* |
| **FG582** *odr-1(n1936); udEx316[hsp::odr-1, elt-2p::gfp]* |
| **FG583** *odr-1(n1936); udEx317[hsp::odr-1, elt-2p::gfp]* |
| **FG584** *odr-1(n1936); udEx318[hsp::odr-1, elt-2p::gfp]* |
| **FG677** *N2; udEx403[hsp::inx-4, elt-2p::gfp]* |
| **FG678** *N2; udEx404[hsp::inx-4, elt-2p::gfp]* |
| **FG679** *N2; udEx405[hsp::inx-4, elt-2p::gfp]* |
| **FG674** *inx-4(ok2373); udEx400[hsp::inx-4, elt-2p::gfp]* |
| **FG675** *inx-4(ok2373); udEx401[hsp::inx-4, elt-2p::gfp]* |
| **FG676** *inx-4(ok2373); udEx402[hsp::inx-4, elt-2p::gfp]* |
| **FG557** *odr-1(n1936); udEx304[str-1p::odr-1, elt-2p::gfp]* |
| **FG558** *odr-1(n1936); udEx305[str-1p::odr-1, elt-2p::gfp]* |
| **FG559** *odr-1(n1936); udEx306[str-1p::odr-1, elt-2p::gfp]* |
| **FG560** *odr-1(n1936); udEx307[ceh-36p3::odr-1, elt-2p::gfp]* |
| **FG561** *odr-1(n1936); udEx308[ceh-36p3::odr-1, elt-2p::gfp]* |
| **FG562** *odr-1(n1936); udEx309[ceh-36p3::odr-1, elt-2p::gfp]* |
| **FG600** *odr-1(n1936); udEx325[gpa-4p::odr-1, elt-2p::gfp]* |
| **FG601** *odr-1(n1936); udEx326[gpa-4p::odr-1, elt-2p::gfp]* |
| **FG602** *odr-1(n1936); udEx327[gpa-4p::odr-1, elt-2p::gfp]* |
| **FG485** *odr-1(n1936); udEx276[srbc-66p::odr-1, elt-2p::gfp]* |
| **FG490** *odr-1(n1936); udEx214[srbc-66p::odr-1, elt-2p::gfp]* |
| **FG491** *odr-1(n1936); udEx215[srbc-66p::odr-1, elt-2p::gfp]* |
| **FG622** *odr-1(n1936); udEx232[trx-1p::odr-1, elt-2p::gfp]* |
| **FG623** *odr-1(n1936); udEx231[trx-1p::odr-1, elt-2p::gfp]* |
| **FG624** *odr-1(n1936); udEx230[trx-1p::odr-1, elt-2p::gfp]* |
| **FG657** *odr-1(n1936); udEx379[str-1p::odr-1, ceh-36p3::odr-1, elt-2p::gfp]* |
| **FG658** *odr-1(n1936); udEx380[str-1p::odr-1, ceh-36p3::odr-1, elt-2p::gfp]* |
| **FG659** *odr-1(n1936); udEx381[str-1p::odr-1, ceh-36p3::odr-1, elt-2p::gfp]* |
| **FG606** *odr-1(n1936); udEx328[str-1p::odr-1, gpa-4p::odr-1, elt-2p::gfp]* |
| **FG607** *odr-1(n1936); udEx329[str-1p::odr-1, gpa-4p::odr-1, elt-2p::gfp]* |
| **FG608** *odr-1(n1936); udEx330[str-1p::odr-1, gpa-4p::odr-1, elt-2p::gfp]* |
| **FG609** *odr-1(n1936); udEx331[ceh-36p3::odr-1, gpa-4p::odr-1, elt-2p::gfp]* |
| **FG610** *odr-1(n1936); udEx332[ceh-36p3::odr-1, gpa-4p::odr-1, elt-2p::gfp]* |
| **FG611** *odr-1(n1936); udEx333[ceh-36p3::odr-1, gpa-4p::odr-1, elt-2p::gfp]* |
| **FG597** *odr-1(n1936); udEx322[str-1p::odr-1, ceh-36p3::odr-1, gpa-4p::odr-1, elt-2p::gfp]* |
| **FG598** *odr-1(n1936); udEx323[str-1p::odr-1, ceh-36p3::odr-1, gpa-4p::odr-1, elt-2p::gfp]* |
| **FG599** *odr-1(n1936); udEx324[str-1p::odr-1, ceh-36p3::odr-1, gpa-4p::odr-1, elt-2p::gfp]* |
| **FG585** *odr-1(n1936); udEx244[odr-1p::odr-1, elt-2p::gfp]* |
| **FG586** *odr-1(n1936); udEx245[odr-1p::odr-1, elt-2p::gfp]* |
| **FG587** *odr-1(n1936); udEx246[odr-1p::odr-1, elt-2p::gfp]* |
| **FG588** *odr-1(n1936); udEx243[odr-1p::odr-1(ΔECD), elt-2p::gfp]* |
| **FG589** *odr-1(n1936); udEx242[odr-1p::odr-1(ΔECD), elt-2p::gfp]* |
| **FG590** *odr-1(n1936); udEx241[odr-1p::odr-1(ΔECD), elt-2p::gfp]* |
| **FG695** *inx-4(ok2373); udEx406[inx-4p::inx-4, vha-6p::mCherry]* |
| **FG696** *inx-4(ok2373); udEx407[inx-4p::inx-4, vha-6p::mCherry]* |
| **FG697** *inx-4(ok2373); udEx408[inx-4p::inx-4, vha-6p::mCherry]* |
| **FG625** *inx-4(ok2373); udEx340[osm-10p::inx-4, elt-2p::gfp]* |
| **FG626** *inx-4(ok2373); udEx341[osm-10p::inx-4, elt-2p::gfp]* |
| **FG627** *inx-4(ok2373); udEx342[osm-10p::inx-4, elt-2p::gfp]* |
| **FG643** *inx-4(ok2373); udEx391[srh-142p::inx-4, elt-2p::gfp]* |
| **FG644** *inx-4(ok2373); udEx237[srh-142p::inx-4, elt-2p::gfp]* |
| **FG645** *inx-4(ok2373); udEx392[srh-142p::inx-4, elt-2p::gfp]* |
| **FG663** *inx-4(ok2373); udEx229[srd-10p::inx-4, elt-2p::gfp]* |
| **FG664** *inx-4(ok2373); udEx393[srd-10p::inx-4, elt-2p::gfp]* |
| **FG665** *inx-4(ok2373); udEx394[srd-10p::inx-4, elt-2p::gfp]* |
| **FG628** *inx-4(ok2373); udEx343[osm-10p::inx-4, srh-142p::inx-4, elt-2p::gfp]* |
| **FG629** *inx-4(ok2373); udEx344[osm-10p::inx-4, srh-142p::inx-4, elt-2p::gfp]* |
| **FG630** *inx-4(ok2373); udEx345[osm-10p::inx-4, srh-142p::inx-4, elt-2p::gfp]* |
| **FG792** *inx-4(ok2373); udEx455[odr-1p::inx-4, elt-2p::gfp]* |
| **FG793** *inx-4(ok2373); udEx456[odr-1p::inx-4, elt-2p::gfp]* |
| **FG794** *inx-4(ok2373); udEx457[odr-1p::inx-4, elt-2p::gfp]* |
| **FG795** *odr-1(n1936); udEx409[osm-10p::BlgC, vha-6p::mCherry, pUC19]* |
| **FG796** *odr-1(n1936); udEx410[osm-10p::BlgC, vha-6p::mCherry, pUC19]* |
| **FG797** *odr-1(n1936); udEx411[osm-10p::BlgC, vha-6p::mCherry, pUC19]* |
| **FG798** *odr-1(n1936); udEx415[osm-10p::BlaC, vha-6p::mCherry, pUC19]* |
| **FG616** *inx-4(ok2373); odr-1(n1936); udEx335[odr-1p::odr-1, elt-2p::gfp]* |
| **FG617** *inx-4(ok2373); odr-1(n1936); udEx336[odr-1p::odr-1, elt-2p::gfp]* |
| **FG618** *inx-4(ok2373); odr-1(n1936); udEx334[odr-1p::odr-1, elt-2p::gfp]* |
| **FG619** *inx-4(ok2373); odr-1(n1936); udEx337[str-1p::odr-1, ceh-36p3::odr-1,*  *gpa-4p::odr-1, elt-2p::gfp]* |
| **FG620** *inx-4(ok2373); odr-1(n1936); udEx338[str-1p::odr-1, ceh-36p3::odr-1,*  *gpa-4p::odr-1, elt-2p::gfp]* |
| **FG621** *inx-4(ok2373); odr-1(n1936); udEx339[str-1p::odr-1, ceh-36p3::odr-1,*  *gpa-4p::odr-1, elt-2p::gfp]* |
| **FG716** *inx-4(ok2373); odr-1(n1936); udEx382[inx-4p::inx-4, elt-2p::gfp]* |
| **FG717** *inx-4(ok2373); odr-1(n1936); udEx383[inx-4p::inx-4, elt-2p::gfp]* |
| **FG718** *inx-4(ok2373); odr-1(n1936); udEx384[inx-4p::inx-4, elt-2p::gfp]* |
| **FG637** *inx-4(ok2373); odr-1(n1936); udEx363[osm-10p::inx-4, elt-2p::gfp]* |
| **FG638** *inx-4(ok2373); odr-1(n1936); udEx364[osm-10p::inx-4, elt-2p::gfp]* |
| **FG639** *inx-4(ok2373); odr-1(n1936); udEx365[osm-10p::inx-4, elt-2p::gfp]* |
| **FG634** *inx-4(ok2373); odr-1(n1936); udEx360[osm-10p::inx-4, odr-1p::odr-1, elt-2p::gfp]* |
| **FG635** *inx-4(ok2373); odr-1(n1936); udEx361[osm-10p::inx-4, odr-1p::odr-1, elt-2p::gfp]* |
| **FG636** *inx-4(ok2373); odr-1(n1936); udEx362[osm-10p::inx-4, odr-1p::odr-1, elt-2p::gfp]* |
| **FG719** *inx-4(ok2373); odr-1(n1936); udEx385[inx-4p::inx-4, odr-1p::odr-1, elt-2p::gfp]* |
| **FG720** *inx-4(ok2373); odr-1(n1936); udEx386[inx-4p::inx-4, odr-1p::odr-1, elt-2p::gfp]* |
| **FG721** *inx-4(ok2373); odr-1(n1936); udEx387[inx-4p::inx-4, odr-1p::odr-1, elt-2p::gfp]* |
| **FG722** *inx-4(ok2373); odr-1(n1936); udEx388[inx-4p::inx-4, str-1p::odr-1, ceh-36p3::odr-1,*  *gpa-4p::odr-1, elt-2p::gfp]* |
| **FG723** *inx-4(ok2373); odr-1(n1936); udEx389[inx-4p::inx-4, str-1p::odr-1, ceh-36p3::odr-1,*  *gpa-4p::odr-1, elt-2p::gfp]* |
| **FG724** *inx-4(ok2373); odr-1(n1936); udEx390[inx-4p::inx-4, str-1p::odr-1, ceh-36p3::odr-1,*  *gpa-4p::odr-1, elt-2p::gfp]* |
| **FG640** *inx-4(ok2373); odr-1(n1936); udEx366[osm-10p::inx-4, str-1p::odr-1, ceh-36p3::odr-1,*  *gpa-4p::odr-1, elt-2p::gfp]* |
| **FG641** *inx-4(ok2373); odr-1(n1936); udEx367[osm-10p::inx-4, str-1p::odr-1, ceh-36p3::odr-1,*  *gpa-4p::odr-1, elt-2p::gfp]* |
| **FG642** *inx-4(ok2373); odr-1(n1936); udEx368[osm-10p::inx-4, str-1p::odr-1, ceh-36p3::odr-1,*  *gpa-4p::odr-1, elt-2p::gfp]* |
| **FG680** *lite-1(ce314); udEx409[osm-10p::BlgC, vha-6p::mCherry, pUC19]* |
| **FG681** *lite-1(ce314); udEx410[osm-10p::BlgC, vha-6p::mCherry, pUC19]* |
| **FG682** *lite-1(ce314); udEx411[osm-10p::BlgC, vha-6p::mCherry, pUC19]* |
| **FG683** *lite-1(ce314); udEx412[srb-6p::BlgC, vha-6p::mCherry, pUC19]* |
| **FG684** *lite-1(ce314); udEx413[srb-6p::BlgC, vha-6p::mCherry, pUC19]* |
| **FG685** *lite-1(ce314); udEx414[srb-6p::BlgC, vha-6p::mCherry, pUC19]* |
| **FG686** *lite-1(ce314); udEx415[osm-10p::BlaC, vha-6p::mCherry, pUC19]* |
| **FG687** *lite-1(ce314); udEx416[osm-10p::BlaC, vha-6p::mCherry, pUC19]* |
| **FG688** *lite-1(ce314); udEx417[osm-10p::BlaC, vha-6p::mCherry, pUC19]* |
| **FG689** *lite-1(ce314); udEx418[srb-6p::BlaC, vha-6p::mCherry, pUC19]* |
| **FG690** *lite-1(ce314); udEx419[srb-6p::BlaC, vha-6p::mCherry, pUC19]* |
| **FG691** *lite-1(ce314); udEx420[srb-6p::BlaC, vha-6p::mCherry, pUC19]* |
| **FG727** *lite-1(ce314); udEx449[srh-142p::BlgC, vha-6p::mCherry, pUC19]* |
| **FG728** *lite-1(ce314); udEx450[srh-142p::BlgC, vha-6p::mCherry, pUC19]* |
| **FG729** *lite-1(ce314); udEx451[srh-142p::BlgC, vha-6p::mCherry, pUC19]* |
| **FG730** *lite-1(ce314); udEx452[srh-142p::BlaC, vha-6p::mCherry, pUC19]* |
| **FG731** *lite-1(ce314); udEx453[srh-142p::BlaC, vha-6p::mCherry, pUC19]* |
| **FG732** *lite-1(ce314); udEx454[srh-142p::BlaC, vha-6p::mCherry, pUC19]* |
| **FG739** *inx-4(ok2373); lite-1(ce314); udEx349[srh-142p::BlgC, vha-6p::mCherry, pUC19]* |
| **FG740** *inx-4(ok2373); lite-1(ce314); udEx350[srh-142p::BlgC, vha-6p::mCherry, pUC19]* |
| **FG741** *inx-4(ok2373); lite-1(ce314); udEx351[srh-142p::BlgC, vha-6p::mCherry, pUC19]* |
| **FG742** *inx-4(ok2373); lite-1(ce314); udEx352[srh-142p::BlaC, vha-6p::mCherry, pUC19]* |
| **FG743** *inx-4(ok2373); lite-1(ce314); udEx353[srh-142p::BlaC, vha-6p::mCherry, pUC19]* |
| **FG744** *inx-4(ok2373); lite-1(ce314); udEx354[srh-142p::BlaC, vha-6p::mCherry, pUC19]* |
| **FG521** *N2; udEx211[osm-10p::ced-3(p15), srb-6p::ced-3(p17), osm-10p::mCherry,elt-2p::gfp]* |
| **FG522** *N2; udEx212[osm-10p::ced-3(p15), srb-6p::ced-3(p17), osm-10p::mCherry, elt-2p::gfp]* |
| **FG523** *N2; udEx213[osm-10p::ced-3(p15), srb-6p::ced-3(p17), osm-10p::mCherry, elt-2p::gfp]* |
| **FG563** *N2; udEx236[str-1p::ced-3(p15), str-1p::ced-3(p17), str-1p::gfp, elt-2p::gfp]* |
| **FG564** *N2; udEx310[str-1p::ced-3(p15), str-1p::ced-3(p17), str-1p::gfp, elt-2p::gfp]* |
| **FG565** *N2; udEx311[str-1p::ced-3(p15), str-1p::ced-3(p17), str-1p::gfp, elt-2p::gfp]* |
| **FG591** *N2; oyIs85 [ceh-36p3::TU#813, ceh-36p3::TU#814, srtx-1p::gfp, unc-122p::dsRed];*  *udEx234[str-1p::ced-3(p15), str-1p::ced-3(p17), str-1p::gfp, elt-2p::gfp]* |
| **FG592** *N2; oyIs85 [ceh-36p3::TU#813, ceh-36p3::TU#814, srtx-1p::gfp, unc-122p::dsRed];*  *udEx233[str-1p::ced-3(p15), str-1p::ced-3(p17), str-1p::gfp, elt-2p::gfp]* |
| **FG593** *N2; oyIs85 [ceh-36p3::TU#813, ceh-36p3::TU#814, srtx-1p::gfp, unc-122p::dsRed];*  *udEx235[str-1p::ced-3(p15), str-1p::ced-3(p17), str-1p::gfp, elt-2p::gfp]* |
| **FG594** *N2; oyIs84 [gpa-4p::TU#813, gcy-27p::TU#814, gcy-27p::gfp, unc-122p::dsRed];*  *udEx319[str-1p::ced-3(p15), str-1p::ced-3(p17), str-1p::gfp, elt-2p::gfp]* |
| **FG595** *N2; oyIs84 [gpa-4p::TU#813, gcy-27p::TU#814, gcy-27p::gfp, unc-122p::dsRed];*  *udEx320[str-1p::ced-3(p15), str-1p::ced-3(p17), str-1p::gfp, elt-2p::gfp]* |
| **FG596** *N2; oyIs84 [gpa-4p::TU#813, gcy-27p::TU#814, gcy-27p::gfp, unc-122p::dsRed];*  *udEx321[str-1p::ced-3(p15), str-1p::ced-3(p17), str-1p::gfp, elt-2p::gfp]* |
| **FG603** *N2; oyIs84 [gpa-4p::TU#813, gcy-27p::TU#814, gcy-27p::gfp, unc-122p::dsRed];*  *oyIs85 [ceh-36p3::TU#813, ceh-36p3::TU#814, srtx-1p::gfp, unc-122p::dsRed];*  *udEx238[str-1p::ced-3(p15), str-1p::ced-3(p17), str-1p::gfp, elt-2p::gfp]* |
| **FG604** *N2; oyIs84 [gpa-4p::TU#813, gcy-27p::TU#814, gcy-27p::gfp, unc-122p::dsRed];*  *oyIs85 [ceh-36p3::TU#813, ceh-36p3::TU#814, srtx-1p::gfp, unc-122p::dsRed];*  *udEx239[str-1p::ced-3(p15), str-1p::ced-3(p17), str-1p::gfp, elt-2p::gfp]* |
| **FG605** *N2; oyIs84 [gpa-4p::TU#813, gcy-27p::TU#814, gcy-27p::gfp, unc-122p::dsRed];*  *oyIs85 [ceh-36p3::TU#813, ceh-36p3::TU#814, srtx-1p::gfp, unc-122p::dsRed];*  *udEx240[str-1p::ced-3(p15), str-1p::ced-3(p17), str-1p::gfp, elt-2p::gfp]* |
| **FG536** *N2; udEx223[gcy28dp::ced-3(p15), gcy-28dp::ced-3(p17), gcy-28dp::gfp, elt-2p::gfp]* |
| **FG537** *N2; udEx224[gcy28dp::ced-3(p15), gcy-28dp::ced-3(p17), gcy-28dp::gfp, elt-2p::gfp]* |
| **FG538** *N2; udEx225[gcy28dp::ced-3(p15), gcy-28dp::ced-3(p17), gcy-28dp::gfp, elt-2p::gfp]* |
| **FG539** *N2; udEx427[srh-124p::ced-3(p15), srh-142p::ced-3(p17), srh-142p::gfp, elt-2p::gfp]* |
| **FG540** *N2; udEx428[srh-124p::ced-3(p15), srh-142p::ced-3(p17), srh-142p::gfp, elt-2p::gfp]* |
| **FG541** *N2; udEx429[srh-124p::ced-3(p15), srh-142p::ced-3(p17), srh-142p::gfp, elt-2p::gfp]* |
| **FG692** *N2; udEx424[gcy-8p::ced-3(p15), gcy-28dp::ced-3(p15), srh-142p::ced-3(p15),*  *aex-3p::ced-3(p17), aex-3p::gfp]* |
| **FG693** *N2; udEx425[gcy-8p::ced-3(p15), gcy-28dp::ced-3(p15), srh-142p::ced-3(p15),*  *aex-3p::ced-3(p17), aex-3p::gfp]* |
| **FG694** *N2; udEx426[gcy-8p::ced-3(p15), gcy-28dp::ced-3(p15), srh-142p::ced-3(p15),*  *aex-3p::ced-3(p17), aex-3p::gfp]* |
| **FG710** *N2; udEx443[str-1p::unc-13 siRNA; elt-2p::gfp]* |
| **FG711** *N2; udEx444[str-1p::unc-13 siRNA; elt-2p::gfp]* |
| **FG712** *N2; udEx445[str-1p::unc-13 siRNA; elt-2p::gfp]* |
| **FG660** *N2; udEx430[ceh-36p3::unc-13 siRNA; elt-2p::gfp]* |
| **FG661** *N2; udEx431[ceh-36p3::unc-13 siRNA; elt-2p::gfp]* |
| **FG662** *N2; udEx432[ceh-36p3::unc-13 siRNA; elt-2p::gfp]* |
| **FG704** *N2; udEx440[gpa-4p::unc-13 siRNA; elt-2p::gfp]* |
| **FG705** *N2; udEx441[gpa-4p::unc-13 siRNA; elt-2p::gfp]* |
| **FG706** *N2; udEx442[gpa-4p::unc-13 siRNA; elt-2p::gfp]* |
| **FG671** *N2; udEx227[odr-1p::unc-13 siRNA; gpa-4p::unc-13 siRNA; elt-2p::gfp]* |
| **FG672** *N2; udEx226[odr-1p::unc-13 siRNA; gpa-4p::unc-13 siRNA; elt-2p::gfp]* |
| **FG673** *N2; udEx228[odr-1p::unc-13 siRNA; gpa-4p::unc-13 siRNA; elt-2p::gfp]* |
| **FG701** *N2; udEx437[gcy-8p::unc-13 siRNA; vha-6p::mCherry]* |
| **FG702** *N2; udEx438[gcy-8p::unc-13 siRNA; vha-6p::mCherry]* |
| **FG703** *N2; udEx439[gcy-8p::unc-13 siRNA; vha-6p::mCherry]* |
| **FG713** *N2; udEx446[gcy-28dp::unc-13 siRNA; vha-6p::mCherry]* |
| **FG714** *N2; udEx447[gcy-28dp::unc-13 siRNA; vha-6p::mCherry]* |
| **FG715** *N2; udEx448[gcy-28dp::unc-13 siRNA; vha-6p::mCherry]* |
| **FG698** *N2; udEx434[srh-142p::unc-13 siRNA; vha-6p::mCherry]* |
| **FG699** *N2; udEx435[srh-142p::unc-13 siRNA; vha-6p::mCherry]* |
| **FG700** *N2; udEx436[srh-142p::unc-13 siRNA; vha-6p::mCherry]* |
